# A secreted WY-domain-containing protein present in European isolates of the oomycete *Plasmopara viticola* induces cell death in grapevine and tobacco species

**DOI:** 10.1371/journal.pone.0220184

**Published:** 2019-07-29

**Authors:** Maud Combier, Edouard Evangelisti, Marie-Christine Piron, David Rengel, Ludovic Legrand, Liron Shenhav, Olivier Bouchez, Sebastian Schornack, Pere Mestre

**Affiliations:** 1 SVQV, Université de Strasbourg, INRA, Colmar, France; 2 University of Cambridge, Sainsbury Laboratory (SLCU), Cambridge, United Kingdom; 3 LIPM Laboratoire des Interactions Plantes-Microorganismes, Université de Toulouse, INRA, CNRS, Castanet-Tolosan, France; 4 US 1426 GeT-PlaGe, Genotoul, INRA, Castanet-Tolosan, France; Agriculture and Agri-Food Canada, CANADA

## Abstract

*Plasmopara viticola* is a biotrophic oomycete pathogen causing grapevine downy mildew. We characterized the repertoire of *P*. *viticola* effector proteins which may be translocated into plants to support the disease. We found several secreted proteins that contain canonical dEER motifs and conserved WY-domains but lack the characteristic RXLR motif reported previously from oomycete effectors. We cloned four candidates and showed that one of them, Pv33, induces plant cell death in grapevine and *Nicotiana* species. This activity is dependent on the nuclear localization of the protein. Sequence similar effectors were present in seven European, but in none of the tested American isolates. Together our work contributes a new type of conserved *P*. *viticola* effector candidates.

## Introduction

Effectors are proteins produced by phytopathogens to manipulate host plants in support of infection. They exert their function by modifying plant physiology and nutrient absorption, as well as by suppressing plant defences [1]. Plants perceive pathogens through recognition of microbial molecular signatures, called PAMPs/MAMPs (Pathogen/Microbe-Associated-Molecular-Pattern) and initiate defence responses in the infection area. Plants also perceive the disturbances caused by penetration and release DAMPs (Damage-Associated -Molecular-Patterns) [2]. Several pathogen effector proteins can interfere with these plant perception mechanisms and the initiated defence responses, resulting in a suppressed plant immunity and infection success. Effectors or their activities may however be detected by plant disease resistance proteins, activating effector-triggered immunity often concomitant with an infection-limiting cell death called hypersensitive response (HR). These events are summarized in the zig-zag model for plant-pathogen interactions [3].

Known oomycete effectors are either secreted into the plant apoplast or reach the plant cell cytoplasm. Two main families of cytoplasmic effectors have been described: CRNs (Crinkling and Necrosis Inducing) and RXLR effectors. Both are characterised by the presence of a conserved amino acid motif on their N-termini, LXLFLAK-DWL for CRNs, and RXLR often followed by dEER for RXLRs. Genetic evidence supports the importance of these motifs in host translocation. Their role in host cell uptake, however, is debated [4–7].

RXLR effectors are the best studied family. The RXLR candidate effector repertoire from several plant pathogenic oomycete species has been reported, and numerous studies in different pathosystems described their contribution to suppressing plant immunity and enhancing pathogen virulence [8,9], their subcellular localisation in the plant cell [10] and the molecular targets of their effector function [9,11,12]. Crystal structures obtained for several RXLR effectors identified a common fold in the C-terminus of many RXLR effector proteins, the WY-domain. This domain may appear either as a single domain or in tandem repeats, and it has been found in the C-terminus of 44% of *Phytophthora infestans* and 26% of *H*. *arabidopsidis* RXLR effectors [13].

The RXLR motif is well conserved and defined within *Phytophthora* species; most RXLR effectors from those species present the canonical motif at the N-terminus. However, this does not seem to be the case for downy mildews. Other motifs, more or less reminiscent of the RXLR, have been reported, like the QXLR and KXLR motifs detected in *Pseudoperonospora cubensis* [14] and the GKLR found in effectors from *Bremia lactucae* [15]. Furthermore, ATR5 from *Hyaloperonospora arabidopsidis* possesses an EER motif but appears to lack a particular motif in position of the RXLR [16]. More recently, the existence of proteins with a WY-domain but lacking RXLR motifs has been reported in the secretomes of *Peronospora tabacina* and *Plasmopara halstedii* [17–19].

*Plasmopara viticola* is an obligate biotrophic oomycete causing grapevine downy mildew. It is present worldwide, attacks all plant organs and is mainly controlled with pesticides. Despite the economic importance of grapevine downy mildew, our understanding of this pathosystem is limited, mainly due to the difficulties of manipulating both host plant and pathogen. In recent years there has been an important increase in the availability of genomic [20–22] and transcriptomic [23,24] resources for *P*. *viticola*, leading to the identification of its RXLR candidate effector repertoire [25,22,26]. The putative role of these effectors as suppressors of defence responses and their subcellular localisation have been reported [26,27], and some notable results include the identification of RXLR effectors inducing or suppressing plant immune responses [27,28].

A sound knowledge of the RXLR effector repertoire and function is essential to understand the mechanisms that govern the grape / *P*. *viticola* interaction and ultimately to develop new approaches to control diseases, substituting those based on chemical inputs. While characterizing the *P*. *viticola* effector repertoire, we identified predicted secreted proteins that harbour EER and WY-domains but lack any RXLR or RXLR-like motifs. In absence of an efficient expression system in grapevine, we studied their function predominantly in the well-established transient expression system *N*. *benthamiana*. We found that Pv33 induces cell death in grapevine and *Nicotiana* species in a nuclear localisation dependent manner.

## Results

### Identification of candidate effectors lacking an RXLR motif

**Fig 1:**
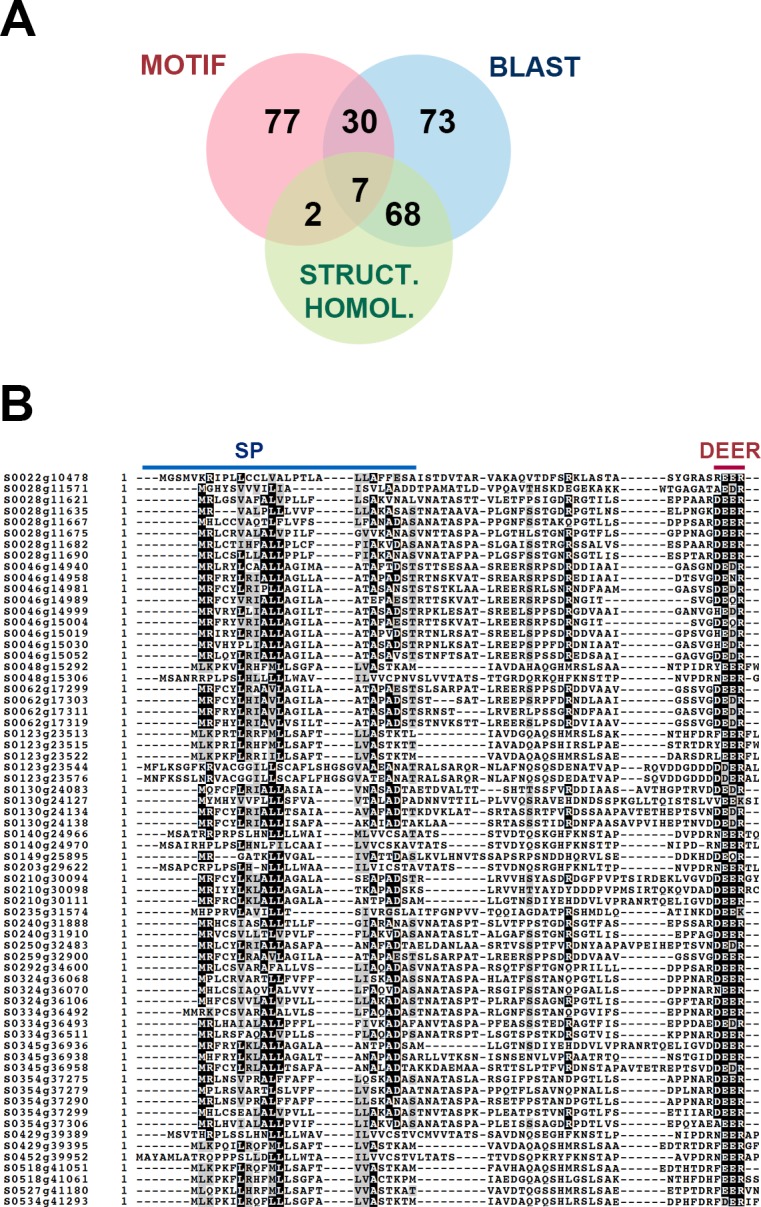
Identification of WY-domain proteins from *P*. *viticola*. (**A**) Venn-diagram showing the distribution of candidate RXLR effectors based on the method leading to their identification and their structural homology to known RXLR effectors. (**B**) Alignment of the N-terminal protein sequences of candidate effector genes showing structural homology to RXLR effectors but lacking the eponymous motif. Signal peptide (SP) and dEER motifs are highlighted.

We searched the *P*. *viticola* proteome for effectors using two strategies: RXLR sequence motif search using string- and HMM-based methods, and low-stringency BLAST-based sequence similarity search against a database of known oomycete RXLR effectors (S2 Dataset). After filtering, we identified a total of 257 primary candidate effector proteins. Of these, 77 effector proteins display structural similarity to known RXLR effectors as determined by Phyre2 (Fig 1A).

Visual inspection of the protein sequences showed that 68 candidates with sequence and structural similarity to RXLR effectors possessed a dEER motif within the first 70 aminoacids, but were lacking a recognisable RXLR motif upstream (Fig 1B and S1 Dataset). Many structurally resolved oomycete effectors carry WY domains and 66 *P*. *viticola* candidates were also predicted to contain at least one WY domain. The numbers of WY domains present in *P*. *viticola* candidates varied between 1 and 10, the most common being 3 detected repeats (22 candidates). We will refer hereafter to these proteins as WY-domain proteins.

To validate the presence of WY-domain proteins from our isolate Pv221 in other *P*. *viticola* isolates we performed BLAST searches against the proteomes of isolates PvitFEM01 [20] and JL-7-2 [22]. The 66 Pv221 WY-domain proteins had significant sequence similarity to 63 and 51 proteins respectively from isolates JL-7-2 and PvitFEM01. With a sequence similarity of greater than 90% we found 52 proteins in JL-7-2, 40 of which having full length matches. In PvitFEM1, 40 matches, with 23 matching full-length sequences.

Using the 66 WY-domain proteins as a query in a low-stringency BLAST against the proteomes of *Phytophthora infestans*, *P*. *parasitica*, *Peronospora tabacina*, *Plasmopara halstedii and Hyaloperonospora arabidopsidis*, we identified candidate secreted proteins containing WY-domain and EER motif but lacking RXLR motifs in all species analyzed (S1 Table and S1 Fig). All proteins, with the exception of 2 from *P*. *halstedii*, showed high confidence structural similarity to RXLR effectors as determined by Phyre2.

### Sequence relationship and expression of *P*. *viticola* WY-domain proteins

**Fig 2:**
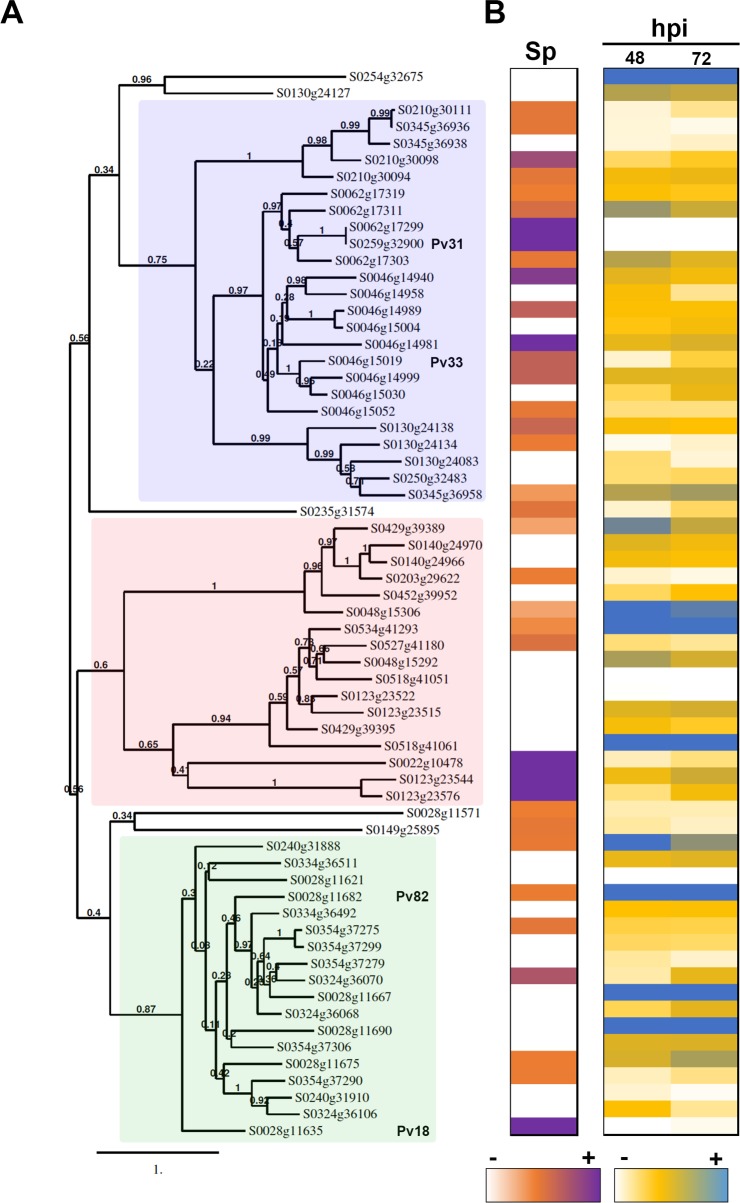
Characterization of *P*. *viticola* WY-domain proteins. **(A)** Phylogenetic tree obtained with the WY-domain proteins from *P*. *viticola*. Branch support values show 100 bootstraps. The three clades referred to in the text are highlighted in pink (I), blue (II) and green (III). Genes selected for further analysis are indicated (**B**) Expression level of WY-domain proteins in germinated spores (Sp) and infected tissues (hpi) at 48- and 72-hours post-infection. White colour shows absence of expression. Detailed expression data is shown in S3 Dataset.

To classify the *P*. *viticola* WY-domain effector candidates further, we constructed a phylogenetic tree which revealed three clades with branch support values higher than 0.6. Each clade contained clusters of sequences belonging to at least two scaffolds, and no sequences from the same scaffold were found in different clades (Fig 2).

We then studied the expression of the WY-domain genes upon infection and in germinated spores. For studying expression upon infection, we used the Illumina RNA-Seq data described in [29], at 48 and 72 hours post-infection. Analysis of expression in germinated spores was performed using the transcriptome described in [24], obtained with Roche 454 GS-FLX Titanium. Most genes were expressed in at least one condition (Fig 2). Most genes from Clade I were expressed to some extent in germinated spores, whilst the majority of genes from Clades II and III were not expressed or weakly expressed at this point. Expression in infected tissues could be detected for 60 out of the 68 genes. Detailed expression data is shown in S3 Dataset.

### A WY-domain effector candidate triggers cell-death in grapevine leaves

**Fig 3:**
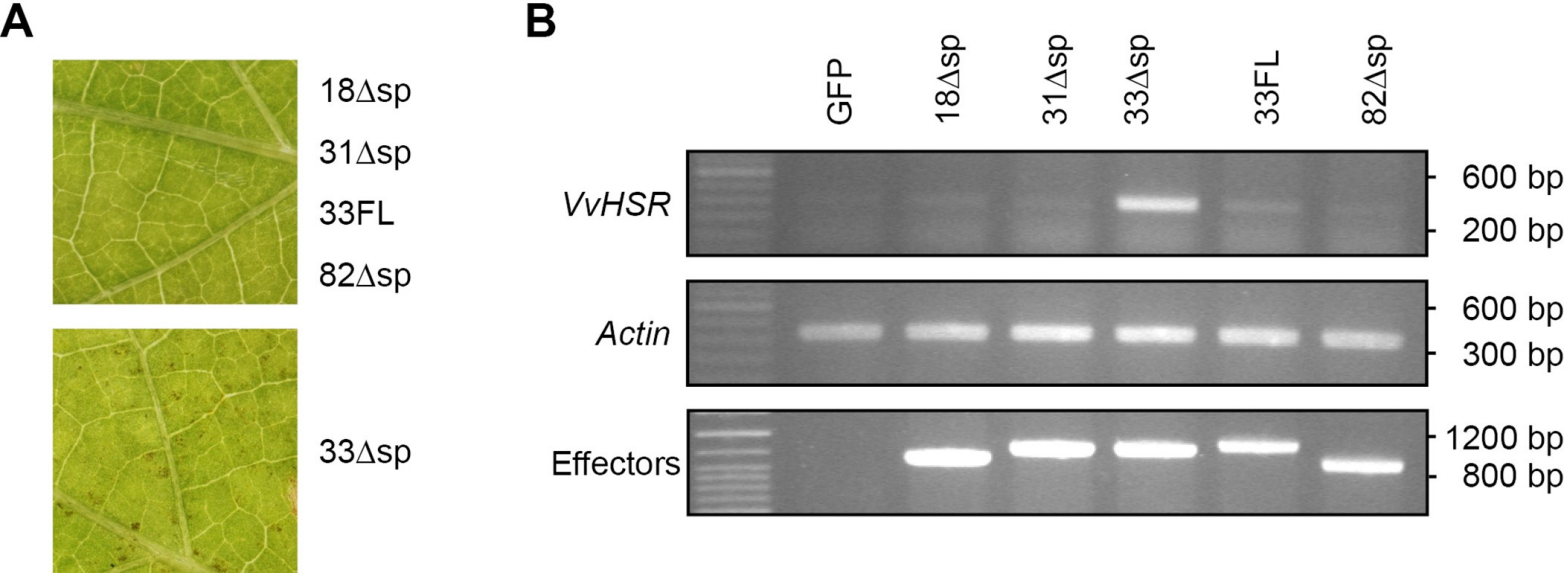
Pv33 induces cell death in *V*. *vinifera* cv Syrah. Agrobacterium-mediated transient expression of Pv18, Pv31, Pv33, Pv82 without their signal peptide (Δsp) and full-length Pv33, including its signal peptide (FL) in *V*. *vinifera* Syrah leaf disks. **(A)** Macroscopic results. Pictures were taken 6 days after agroinfiltration and are representative of the phenotype of each effector. **(B)** Semi-quantitative RT-PCR showing *VvHSR* and effector expression. *Actin* expression is used as reference. Agrobacterium-mediated transient expression of GFP was used to confirm that the induction of *VvHSR* expression is specific to Pv33. Each effector-expressing Agrobacterium strain was infiltrated into 4 leaf discs and the experiment was repeated twice. The macroscopic effect of 33Δsp was not visible in all infiltrated discs but could be observed in both repetitions. RNA extractions for RT-PCRs were done by pooling the 4 disks for each effector.

To study the function of WY-domain proteins we selected and cloned four WY-domain proteins showing high expression level either in germinated spores or upon infection, Pv18 (S0028g11635; 3 WY-domain repeats), Pv31 (S0259g32900; 2 WY-domain repeats), Pv33 (S0046g15019; 1 WY-domain repeat), Pv82 (s0028g11682; 3 WY-domain repeats) without their secretion peptide, which we named Pv18Δsp, Pv31Δsp, Pv33Δsp and Pv82Δsp. To observe their effect in grapevine, the *P*. *viticola* host plant, we transiently expressed the above-described effectors in leaf discs of *V*. *vinifera* cv. Syrah. The efficiency of transformation was low, but macroscopic cell death was visible in 2 out of 4 leaf discs infiltrated with *Agrobacterium* containing Pv33Δsp (Fig 3A). Cell death was not observed in any of the leaf discs infiltrated with the other 3 effector candidates or with Pv33FL, a full-length version of Pv33 including its signal peptide. Concomitantly, we detected induction of the expression of the cell death marker *VvHSR1* [30–32] in leaf discs transiently expressing Pv33Δsp, which could not be detected in leaf discs transiently expressing the other constructs (Fig 3B). The results obtained with Pv33Δsp were confirmed by transiently expressing the effector in whole leaves using a needleless syringe (S2 Fig). Together, these results show that the WY-domain protein Pv33Δsp induces cell death in grapevine leaves.

### Pv33 is present in several European isolates, expressed during grape infection

We next investigated if Pv33 was broadly distributed in *P*. *viticola* populations. To do so, we performed PCRs with primers spanning from ATG to STOP on a set of isolates from different geographic origins. We obtained PCR products from all 7 European isolates tested, but not from isolates from North America (S2 Table). Sequencing of the PCR products revealed low amino acid sequence variability (S3 Fig). These results show that Pv33 is present in European populations.

**Fig 4:**
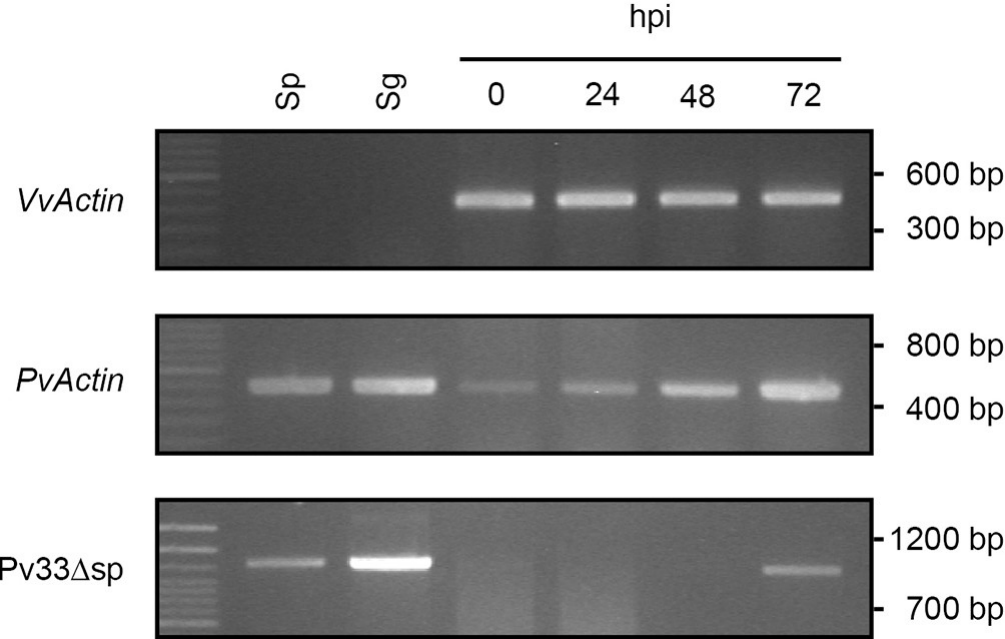
Expression of Pv33 in *P*. *viticola* developmental stages. Semi-quantitative RT-PCR of Pv33 expression in sporangia (Sp), germinated spores (Sg) and infected tissues at 0, 24, 48 and 72 hours after inoculation (hpi). Expression of *V*. *vinifera* Actin (*VvActin*) is shown as loading of samples corresponding to infected tissues. Expression of *P*. *viticola* Actin (*PvActin*) reflects pathogen biomass and shows progression of infection.

To understand where in the *P*. *viticola* infection cycle would Pv33 be playing a role, we used semi-quantitative RT-PCR to validate the expression of Pv33 in different stages of *P*. *viticola* development in grapevine including sporangia, germinated spores, and infected grapevine tissues at different time-points after inoculation. Pv33 expression could be observed in sporangia (Sp) germinated spores (Sg) and infected tissues at 72 hours post-infection (hpi) (Fig 4). Expression levels were highest in germinated spores, suggesting a role early in infection.

### Pv33Δsp induces cell death in different *Nicotiana* species

**Fig 5:**
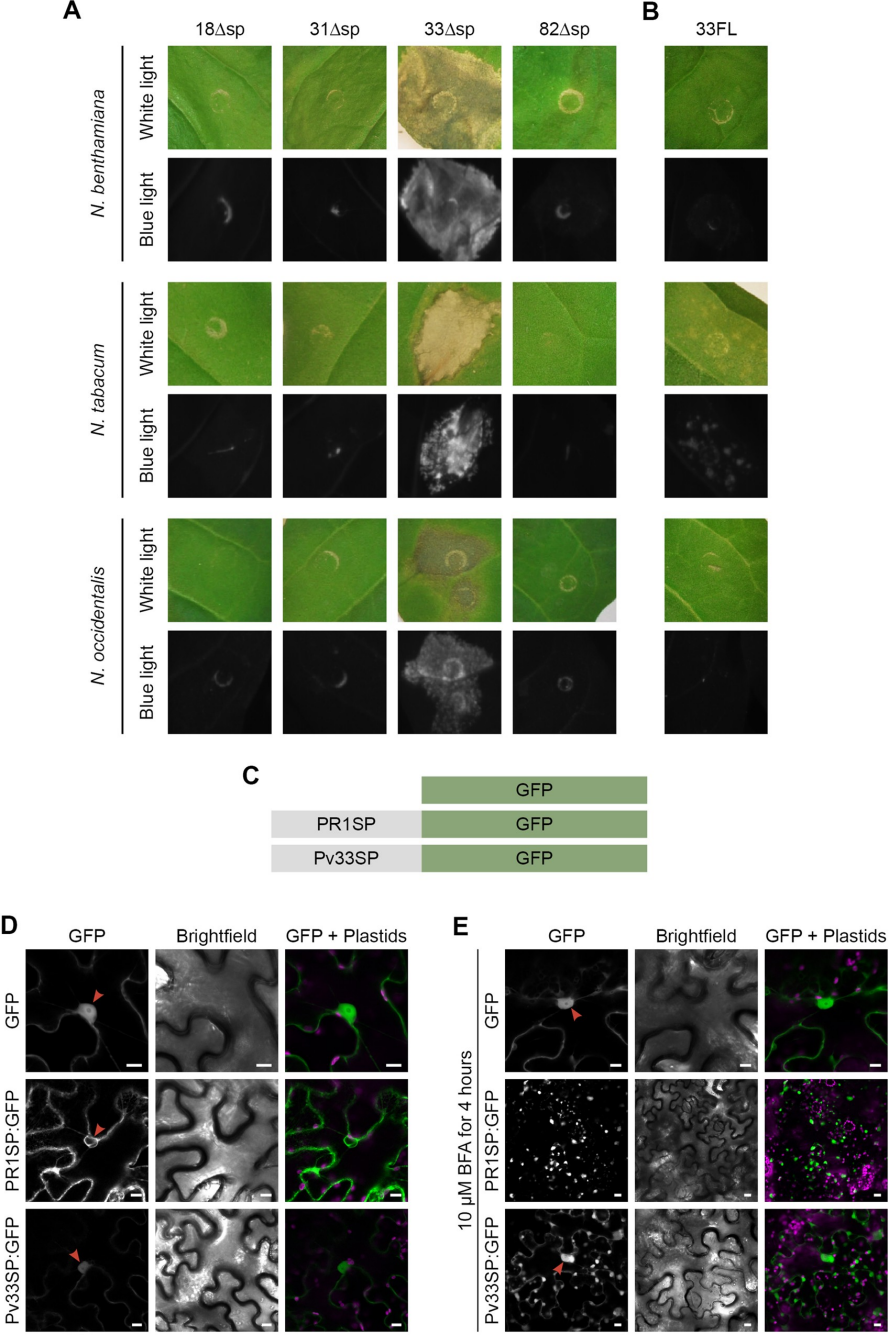
Pv33 induces cell death in *Nicotiana spp*. Agrobacterium-mediated transient expression of Pv18, Pv31, Pv33, Pv82 without their signal peptide (Δsp) **(A)** and full-length Pv33, including its signal peptide (FL) **(B)** in *N*. *benthamiana*, *N*. *tabacum* and *N*. *occidentalis* leaves. All pictures were taken 5 days after agroinfiltration. **(C)** Constructs used in experiments shown in D and E. PR1SP: signal peptide of PR1. Pv33SP: signal peptide of Pv33. **(D and E)** Confocal images following infiltration of *N*. *benthamiana* leaves with the constructs described in C, without **(D)** and with **(E)** BFA treatment. Red arrows show nucleus. Bars show 10 Δm. In A and B, for each species, 6 leaves were infiltrated with all the effectors. Experiments were repeated twice with the same results.

Due to the limitations of the grapevine transient expression system to perform further studies with Pv33, we verified if Pv33Δsp would also induce cell death in *Nicotiana* species. Pv33Δsp was able to induce cell death in *Nicotiana benthamiana*, *N*. *occidentalis* and *N*. *tabacum* (Fig 5A) following *Agrobacterium*-mediated transient expression. Pv33FL did not induce cell death in *N*. *benthamiana* and *N*. *occidentalis* and only a few necrotic spots in *N*. *tabacum* (Fig 5B), confirming the results obtained with grapevine and suggesting that cytoplasmic expression is needed for the Pv33 cell death activity.

In parallel, to study the possible role of WY-domain proteins as suppressors of plant defences we studied their ability to suppress the INF1-induced cell death upon *Agrobacterium*-mediated transient expression in *Nicotiana benthamiana* [33]. None of the proteins was able to suppress the INF1-mediated cell death (S4 Fig).

We next investigated the function of the signal peptide of Pv33 by fusing it to GFP and expressing it in leaves of *N*. *benthamiana*. Native GFP and GFP fused to the signal peptide of PR1 were used as negative and positive controls respectively. The fluorescence observed after transient expression of Pv33SP:GFP showed a pattern different to that observed with PR1SP:GFP, with weaker intensity and residual nucleoplasmic fluorescence (Fig 5D). Following BFA treatment, which blocks vesicular traffic, typical BFA bodies could be observed with both PR1SP:GFP and Pv33SP:GFP, with nuclear fluorescence still visible for Pv33SP:GFP (Fig 5E). The nuclear fluorescence observed with Pv33SP:GFP shows that part of the protein is not incorporated in the secretory pathway, suggesting that the signal peptide of Pv33 is only partially functional in plant cells, potentially explaining the retained cell death inducing activity of Pv33FL in *N*. *tabacum*.

### Pv33Δsp-mediated HR is SGT1-dependent and EDS1-independent

**Fig 6:**
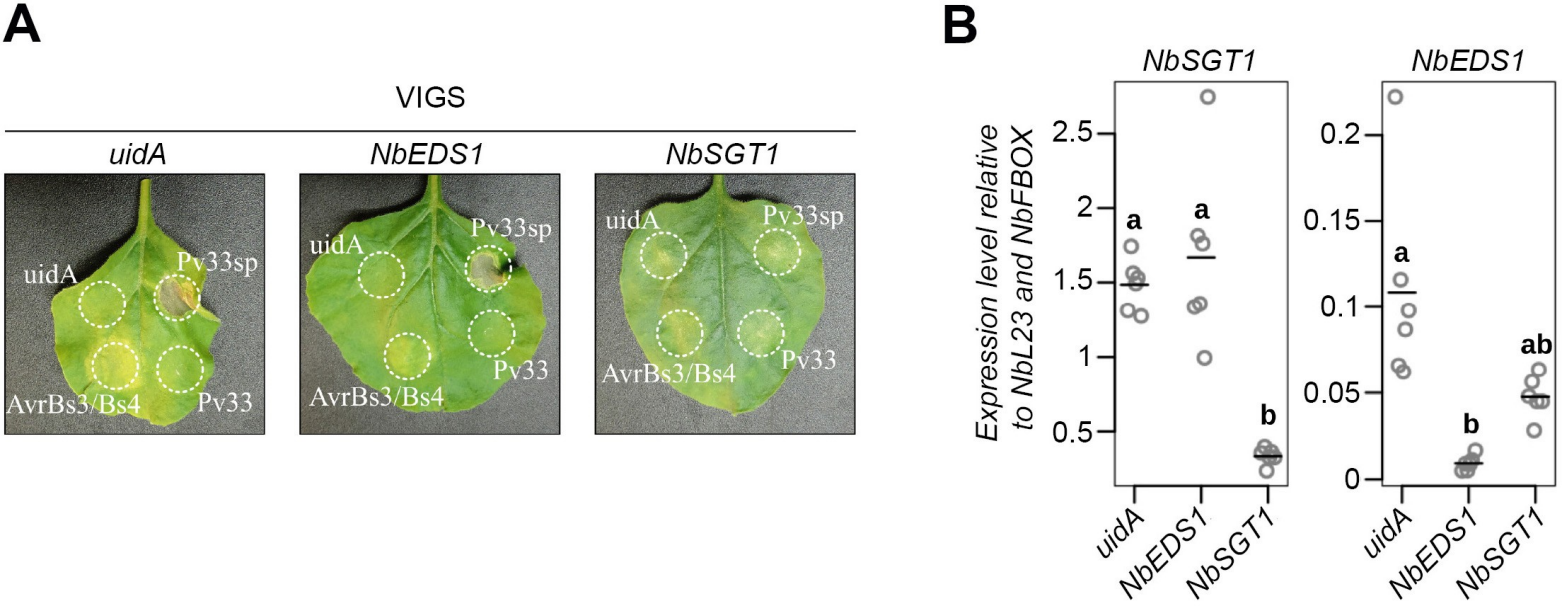
Pv33-triggered cell death requires SGT1. **(A)** Representative pictures of cell death assays on TRV-silenced leaves. Pictures were taken 5 days after agroinfiltreation with *A*. *tumefaciens* cells carrying a construct for constitutive expression of uidA (GUS), 33 full length (33FL) or without signal peptide (33Δsp), or a 1:1 combination of *Xanthomonas* effector AvrBs3 and the resistance gene Bs4, used as positive control. The experiment was performed in quadruplicate (N = 32). **(B)** Validation of virus-induced gene silencing by quantitative RT-PCR. Accumulation of transcripts from *N*. *benthamiana* genes *EDS1* and *SGT1* in 6 individual plants, 3 weeks after agroinfiltration with *A*. *tumefaciens* cells carrying a construct for TRV-induced gene silencing of *uidA*, *EDS1* and *SGT1*. Data are shown relative to *L23* and *F-BOX* reference genes. Circles represent ratios, lines represent means. Statistical significance was assessed using one-way ANOVA and Tukey’s HSD test (P < 0.05).

In order to shed light in the nature of the Pv33Δsp-induced cell death, we used Virus-Induced Gene Silencing (VIGS) to study the role of *SGT1* and *EDS1* in this response. *EDS1* is required for the HR mediated by the TIR-NBS-LRR immune receptors [34,35], whilst *SGT1* is required for the cell death mediated by different plant-pathogen interactions [36]. SGT1 silencing resulted in mild developmental symptoms (S5 Fig). Transient expression of Pv33Δsp on *N*. *benthamiana* silenced for *EDS1* and *SGT1* resulted in the induction of cell death in EDS1-silenced plants and strong reduction of cell death in *SGT1*-silenced plants (Fig 6A). The silencing efficiency was monitored by quantitative RT-PCR (Fig 6B). These results indicate that the Pv33Δsp-triggered cell death requires *SGT1* but it does not involve *EDS1* and thus is likely not mediated by a TIR-NBS-LRR type disease resistance protein.

### 33Δsp localizes to the nucleus and the localization is required for HR induction

**Fig 7:**
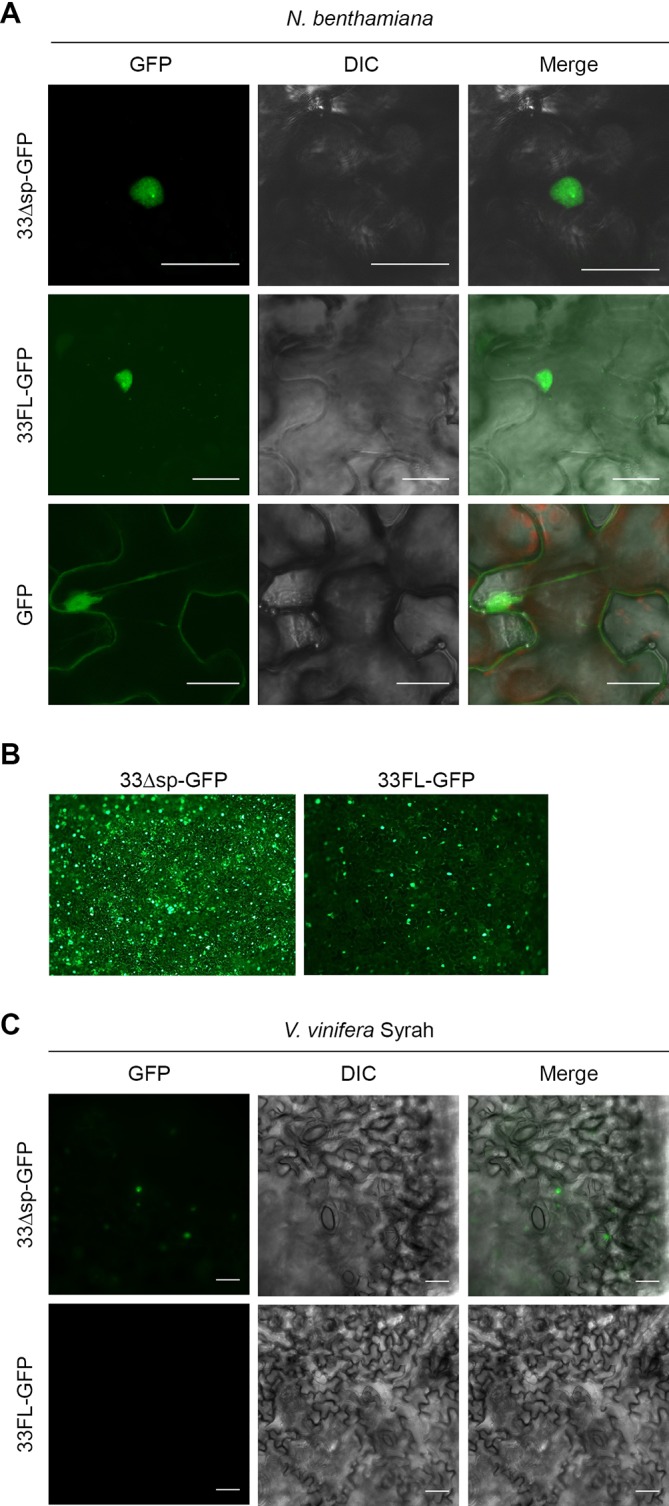
Pv33 localizes to the nucleus. **(A)** Confocal microscopy images following infiltration of *N*. *benthamiana* leaves with Agrobacterium carrying GFP translational fusions of Pv33 either lacking (33Δsp-GFP) or including (33FL-GFP) the signal peptide. GFP alone was used as control. **(B)** Low magnification epifluorescence microscopy images of 33ΔSP-GFP and 33FL-GFP infiltrations. **(C)** Epifluorescence microscopy images following infiltration of *V*. *vinifera* cv Syrah leaf discs with Agrobacterium carrying 33ΔSP-GFP and 33FL-GFP. Pictures were taken 2 days after agroinfiltration. Experiments were repeated 5 times for *N*. *benthamiana* and twice for *V*. *vinifera*, with the same results. Bar shows 25 µm.

To study cellular localization, we obtained GFP translational fusions of Pv33Δsp and Pv33FL. Transient expression of Pv33Δsp-GFP in *N*. *benthamiana* resulted in the fluorescence signal localized to the nucleus, and the signal appeared enhanced in the nucleolus (Fig 7A). Leaves transiently expressing Pv33FL-GFP displayed an overall lower level of fluorescence (Fig 7B); nuclear fluorescence was reduced and fluorescent spots appeared in the periphery of the epidermal cells (Fig 7A). Similar results were obtained with Pv33ΔSP-GFP in *V*. *vinifera* (Fig 7C), where the general lower expression level hindered us to observe any fluorescence in leaf discs transiently expressing Pv33FL-GFP.

We next investigated if Pv33 contained a nuclear localisation signal (NLS). Search for NLS features using NLStradamus [37], NLS Mapper [38], NucPred [39], Psort [40], Predict NLS [41] and SeqNLS [42] didn’t predict any canonical NLS.

**Fig 8:**
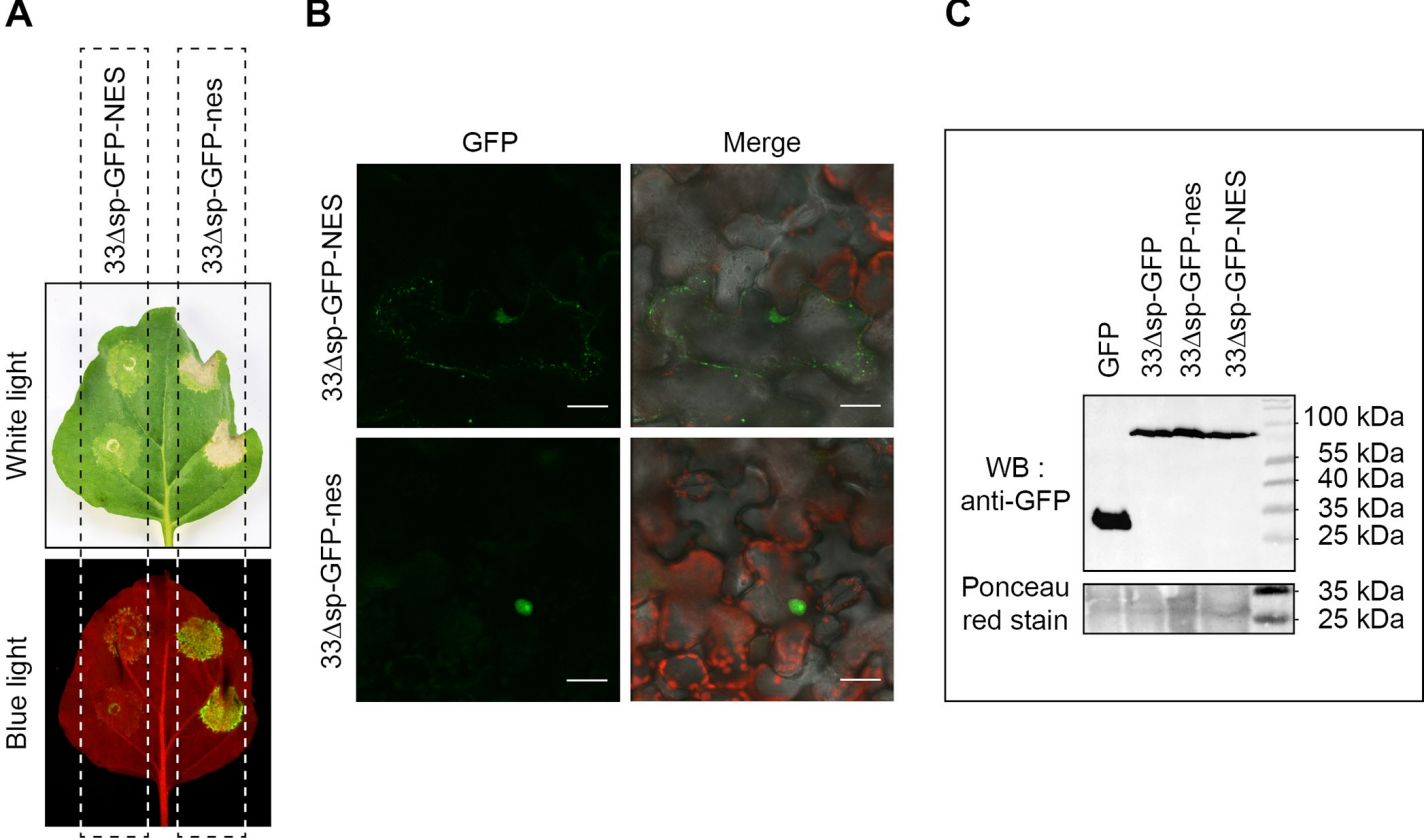
Pv33-triggered cell death depends on its nuclear localisation. **(A)** Agrobacterium-mediated transient expression of *N*. *benthamiana* leaves with 33Δsp-GFP fused to either an active (33Δsp-GFP-NES) or inactive (33Δsp-GFP-nes) nuclear exclusion sequence. Pictures were taken 5 days after agroinfiltration under white and blue light. **(B)** Subcellular localisation of 33Δsp-GFP-NES and 33Δsp-GFP-nes as observed by confocal microscopy 2 days after agroinfiltration. **(C)** Western blot with anti-GFP of the different GFP-tagged Pv33 versions. GFP is used as positive control. Ponceau red staining of the nitrocellulose membrane is shown as loading marker. Results shown in A and B are representative of 3 independent experiments, each consisting of at least 6 *N*. *benthamiana* leaves infiltrated as shown in A.

To further study the role of nuclear localization in the induction of cell death by Pv33Δsp, we obtained translational fusions of Pv33Δsp-GFP containing a Nuclear Export Signal (NES) and a mutated, inactive nuclear export signal (nes). Pv33Δsp-GFP-NES-induced cell death was strongly reduced when transiently expressed in *N*. *benthamiana* leaves and its fluorescence was limited to the peripheral spots, while Pv33Δsp-GFP-nes retained nuclear localization and the full cell death-inducing activity (Fig 8A and 8B). All constructs were expressed to similar levels (Fig 8C). These results indicate that Pv33Δsp-triggered death cell is dependent on its nuclear localization.

## Discussion

In this study we described *Plasmopara viticola* secreted proteins possessing the WY-domain and EER motif characteristic of oomycete RXLR effectors but lacking the eponymous motif. We showed that one of these proteins, named Pv33, induces cell death in grapevine and *Nicotiana* species. Pv33-induced cell death in *N*. *benthamiana* is SGT1-dependent and requires nuclear localization. The fact that Pv33 induced cell death, together with the difficulty of genetic transformation in *P*. *viticola*, precluded us from validating its involvement in the virulence of the pathogen or its role in suppressing plant defences.

Phylogenetic analysis of *P*. *viticola* WY-domain-containing proteins grouped them in three clades. Comparing the phylogeny with the gene’s expression pattern revealed that most genes from Clade I were expressed in germinated spores, and the opposite was true for the other two clades (Fig 2). Also, high levels of expression in infected tissues were observed for members of clades II and III, but never for genes from clade I. These results suggest that clade I might include mostly genes expressed very early upon infection, whilst genes from clades II and III would be expressed mostly later in infection. Different temporal patterns of expression of effectors has been reported for oomycetes [43]. These observations suggest that a specific type of candidate effector sequence may be required at different stages of the pathogen life cycle.

Secreted WY-domain-containing proteins were found in the genome of three *P*. *viticola* isolates from different geographical origins, Pv221 (France, this study), PvitFEM01 (Italy, [20]) JL-7-2 (China, [22]). The differences in protein numbers in each isolate could reflect either the variability between isolates or the differences in completeness of the different genomic assemblies [20]. Similar proteins with EER motif and WY domain but lacking a recognisable RXLR motif were found in all investigated oomycete species genomes (S1 Fig and S1 Table) and had previously been reported from *P*. *tabacina* and *P*. *halstedii* [17–19]. Furthermore, the secretome of *H*. *arabidopsidis* contains 173 proteins that, similar to ATR5, carry an EER motif but lack the RXLR [16]. Our non-exhaustive search for WY-domain-containing proteins in other oomycetes retrieved some of the proteins previously described as such in the secretomes of *P*. *tabacina* and *P*. *halstedii* (S1 Fig). Notably, *P*. *viticola* sequences with high levels of sequence conservation among oomycetes often carried RXLR motifs in their *Phytophthora* homologs. Two examples, including Pv33, are listed in S6 Fig.

The RXLR-dEER motif has been shown to be required for translocation of RXLR effectors inside the plant cell [7]. Research in other oomycetes, particularly the downy mildews, resulted in the identification of several effectors that are translocated inside the plant cell and lack canonical RXLR motifs. In *Pseudoperonospora cubensis*, effectors carrying a QXLR motif were shown to localize to the plant nucleus and induce an HR in *N*. *benthamiana* [14]; effector proteins from *Bremia lactucae* carrying a GKLR motif are recognised inside lettuce plant cells in an R-gene specific manner [15]; ATR5 from *Hyaloperonospora arabidopsidis* is recognised inside Arabidopsis cells by the NLR RPP5 even when it lacks an RXLR motif [16], although it possesses a GRVR sequence at the equivalent position, which remains of the above-described GKLR. All these examples commonly possess an EER motif, suggesting that its presence may in part alleviate the need for a RXLR motif for successful translocation. Examination of the Pv33 sequence, or an alignment of WY-domain proteins from the same clade, did not identify any conserved motif upstream of the EER similar to the above described (S7 Fig).

In some experiments the transient expression of Pv33 containing a signal peptide (Pv33FL) resulted in necrotic spots and mild induction of *VvHSR*. Interestingly, Pv33FL-GFP shows residual nuclear localisation, which could explain the eventual mild induction of cell death by Pv33FL. In this context, the amount of protein localising to the nucleus after transient expression of 33FL would be below the threshold necessary to activate cell death. The residual nuclear fluorescence observed with Pv33FL-GFP would most likely be the result of the signal peptide of Pv33 being inefficiently recognised by the plant secretion system (Fig 5D and 5E).

RNA-Seq data and RT-PCR experiments showed that Pv33 is expressed in sporangia, germinated spores and infected tissues at late infection, with the strongest expression being observed in germinated spores (Fig 4). Germinated spores present a germinative tube and may be considered as representative of the early stages of infection, with expression of effector genes taking place [25]. The expression pattern that we observe could thus fit with Pv33 being expressed at low level all through the *P*. *viticola* life cycle. Alternatively, Pv33 would be expressed only in spores and the expression observed at 72 hpi would be associated with the onset of sporulation. Although we cannot rule out this possibility, the RNA-Seq data showing expression of Pv33 at 48 hpi (too early for the start of sporulation), supported by an RT-PCR with higher number of cycles (S8 Fig), makes us favour the hypothesis of Pv33 being most relevant early upon infection.

Supporting an important role of Pv33 in the life cycle of *P*. *viticola*, we found the gene to be conserved in its amino acid sequence in all 7 tested European isolates making it a potential target for resistance breeding strategies. *P*. *viticola* was introduced into Europe from North America in the late 19th century. It is thus surprising that Pv33 is not present in any of the North American isolates (S2 Table). This could be explained if there was variability in the presence of Pv33 in the original North American populations. For example, Pv33 could be dispensable in North American populations due to functional redundancy with other effectors; a bottleneck effect of the introduction could thus make Pv33 important for the pathogen’s biology if the introduced isolate was missing other effectors with similar functions, or if it made it better adapted to the new host landscape.

Cell death induction by oomycete effectors has been reported previously. *P*. *infestans* RXLR effector PexRD2 has been reported as inducing *SGT1*-dependent cell death in *N*. *benthamiana* [44] and 10 *Solanum* species [45]; *P*. *sojae* Avh241 induces cell death in several plant species [46]; PpE4 from *P*. *parasitica* triggers cell death in Solanaceae [47]; PvRxLR16 from *P*. *viticola* induces cell death in *N*. *benthamiana* that is dependent on its nuclear localisation [28]. Conversely, oomycete CRN effectors were first identified as inducing necrosis when transiently expressed in plants [48]. They are localised to the plant nucleus and nuclear localisation is required for the induction of HR [5]. CRNs are considered as an ancestral class of oomycete effectors, since they are present in all lineages of oomycetes [5] and they can be found in species with different life styles, from necrotrophic [49] to biotrophic [24,50]. It has been hypothesized that the necrosis-inducing effect of CRNs will not reflect their real function in the infection process but it would rather be a “phenotypic manifestation reporting on effector activity” [51], an hypothesis that we favour for Pv33 based on our results.

Pv33 induces cell death both in members of the *Solanaceae* and the *Vitaceae*, which are phylogenetically distant. It seems less likely that the response is mediated by an R-gene mediated mechanisms conserved between both families. Accordingly, silencing of EDS1 did not affect the Pv33-induced cell death, suggesting that no TNL-type R gene is involved. Silencing of NLR-helper genes required for the function of diverse sensor R-genes of the CNL class [52] will help to clarify the eventual involvement of an NLR-type R gene in the cell death induced by Pv33. Independently of the underlying mechanism, the Pv33 induced cell death activity that we observe upon ectopic expression of the protein alone could be suppressed by another *P*. *viticola* effector in the context of an infection, avoiding a negative effect in pathogen development. A similar phenomenon has been reported for the biotrophic nematode *Heterodera avenae*, where the expression of cell death-inducing effectors upon infection does not have a negative effect on the pathogen’s life cycle [53].

In summary, here we identified and characterised Pv33, a signal peptide and WY-domain-containing protein from the oomycete *P*. *viticola* that is nuclear localized when expressed in plants and that induces cell death in plant cells. Xiang et al. [28] reported similar results with PvRxLR16, a canonical RXLR effector. Contrary to PvRxLR16, Pv33 lacks RXLR or other motifs described in oomycetes between the signal peptide and EER motifs and PvRxLR16-mediated cell death is N-glycosylation dependent, whilst our protein lacks any putative N-glycosylation sites. Future research will aim at solidifying the roles of Pv33 and other WY-domain-containing proteins inside plant cells during the infection of grape by *P*. *viticola*.

## Materials and methods

### Plant and pathogen materials

*Vitis vinifera* cv Syrah was grown on potting soil from green cuttings in a greenhouse at 22–19°C (day-night) and a photoperiod of 16/8h (light/ dark). Plants were propagated as cuttings every 3 months.

*Nicotiana benthamiana* was grown in greenhouse on soil at min 18°C- max. 28°C and a photoperiod 14h of light (10 klx min).

*Plasmopara viticola* isolate Pv221 was collected in Blanquefort (France) and maintained on detached leaves from *V*. *vinifera* cv Muscat Ottonel. Germinated spores and infected tissues were obtained as described in [25].

### Sequence analysis

The proteome of *P*. *viticola* isolate Pv221 [21] was used to search for candidate RXLR effectors using the Galaxy workflow described in [54]. Genes identified as putative RXLRs by the method described in [55] and any of the two methods described in [7] were selected. At the same time, the proteome was used as a query in a BLAST search at low stringency (Evalue<10e-5) against a database of oomycete RXLR effectors. The BLAST output was analyzed with SignalP4 and TMHMM 2.0, and proteins lacking a signal peptide or possessing a transmembrane domain outside the first 70 amino acids were discarded. Finally, candidate effector genes were subjected to structural homology searches using Phyre2 (http://www.sbg.bio.ic.ac.uk/phyre2/html/page.cgi?id=index) with normal modeling mode [56]. Candidate genes displaying high confidence (100%) structural homology to proteins of known function and lacking RXLR and/or DEER motifs at the predicted positions were considered as false positives and discarded. WY-domain search was performed with the hmmsearch function of HMMER 3.1b2 using the HMM WY_motif.hmm and a cutoff of ≥ 0.

Alignment in Fig 1B was performed with MUSCLE, manually edited and displayed with BOXshade, with a cutoff of 50% of sequences identical for shading. The alignment shows 66 out of the 68 sequences, since removing 2 of them produced an alignment with less gaps and reduced amount of manual editing required.

Phylogenetic analyses were performed at www.phylogeny.fr [57]. Alignments were performed with Muscle with standard mode and default parameters. Following visual inspection of the alignment, 4 out of the 68 sequences were removed to improve the alignment and thus tree robustness. Curation with GBlocks could not be applied to the sequence set. Tree was constructed using PhyML with default settings and branch support was calculated using 100 bootstrap repetitions.

### Expression data

Expression data in germinated spores was obtained with Roche 454 GS-FLX Titanium and is described in [24]. Germinated spore’s transcriptome data was obtained with strains PvSC and PvSL. To identify expressed genes from Pv221, candidate RXLR genes identified as described above were used as a query in a BLASTN against the transcriptome. Genes showing hits with both identity and subject cover higher than 90% were considered as expressed in germinated spores. The number of reads was counted for each gene and normalized by the gene mRNA size.

Expression data in infected tissues was obtained by RNA-Seq with three biological replicates per time point. RNA extraction and sequencing are described in [29]. RNA-Seq read pairs were mapped on the *P*. *viticola* 221 genome using the glint software with parameters set as follows: matches ≥50 nucleotides, ≤4 mismatches, no gap allowed, only best-scoring hits considered. Ambiguous matches (same best score) were removed. RNA-Seq stats and RSA accession numbers are provided in S3 Table.

Mapped reads were further analyzed under R environment (R Core Team 2018, https://www.R-project.org/) using the edgeR library [58] in order to calculate TMM-normalized Counts per Million mapped reads or cpm. These values were then normalized by the predicted mRNA size.

### Plasmid constructs

*P*. *viticola* genomic DNA was extracted from infected leaves at the sporulation stage using the Qiagen DNeasy plant Mini kit with the following modifications: PVP30 was added to the AP1 buffer (25mg/mL) and elution was performed with buffer preheated at 65°C. The sequence of the *P*. *viticola* effectors was amplified from *P*. *viticola* genomic DNA using primers carrying overhangs with restriction enzymes. Amplifications were performed with Phusion polymerase (NEB). Amplified products were digested with the corresponding restriction enzymes (NEB) and cloned directionally into binary plasmid pBIN61 digested with the same enzymes.

To obtain GFP fusions, effectors were amplified as described above and cloned directionally into the pGFPS65T vector, which corresponds to pGFP [32] carrying the GFP S65T mutation.

Nuclear Exclusion Signal (NES) and mutated nuclear exclusion signal (nes), as defined in [59], were added by amplifying the GFPS65T sequence with primers including the signal sequences and appropriate restriction enzymes, and then replacing the GFP sequence in the pGFPS65T-33Δsp construct with the ones containing respectively the NES and nes sequences.

PR1sp:GFP was amplified from pTrafficLights [60]. Pv33sp:GFP was amplified from GFP using a forward primer containing the sequence of the Pv33 signal peptide. Constructs were cloned into a modified pUB-Dest vector derived from [61] containing a 1500 bp *Arabidopsis* ubiquitin 10 (AtUBQ10) promoter sequence for enhanced expression in *N*. *benthamiana*.

Identity of all clones was confirmed by sequencing. A list of constructs and primer sequences is presented in S4 Table.

### Transient expression of genes by agroinfiltration

Agroinfiltration of *N*. *benthamiana* was performed as described in [62].

To agroinfiltrate *V*. *vinifera* leaves, bacterial suspensions were prepared as described in [63] and Silwet-L77 was added to the bacterial suspension at 0.3% final concentration. Infiltrations were performed on leaf discs derived from young leaves by immerging the discs for 10 minutes in the bacterial solution, as described in [64].

### Virus-Induced Gene Silencing (VIGS)

For VIGS experiments, *Nicotiana benthamiana* was grown on Levington F2 at 24°C with 16 h photoperiod for two weeks prior to agroinfiltration. Two-week-old *N*. *benthamiana* plants were agroinfiltrated with a 1:1 mix of *A*. *tumefaciens* GV3101 cells carrying either TRV1 or a recombinant pYL279a for silencing of *uidA* (negative control), *NbEDS1* or *NbSGT1* and further grown in the same conditions for two more weeks. Then, newly formed leaves were infiltrated with *A*. *tumefaciens* GV3101 cells carrying either *uidA*, *Pv33*, *Pv33*Δ*sp*. A 1:1 mix of *A*. *tumefaciens* GV3101 cells carrying *AvrBs3* and *Bs4* were infiltrated as a control. Symptoms were monitored five days after agroinfiltration.

### Quantitative reverse transcription-polymerase chain reaction (qRT-PCR) analyses

Total RNA was extracted 10 days after VIGS agronfiltration from newly formed leaves using RNeasy Plant Mini Kit (Qiagen, USA). One microgram was reverse transcribed to generate first-strand cDNA, using the Roche Transcriptor First Strand cDNA Synthesis Kit according to the manufacturer’s instructions (Roche, Switzerland). Quality was assessed by electrophoresis on agarose gel. qRT-PCR experiments were performed with 2.5 μl of a 1:20 dilution of first-strand cDNA and LightCycler 480 SYBR Green I Master mix, according to the manufacturer’s instructions (Roche, Switzerland). Gene-specific oligonucleotides were designed with BatchPrimer3 software (http://probes.pw.usda.gov/batchprimer3/) outside of the sequence used for VIGS silencing and their specificity was validated by analyzing dissociation curves after each run. Genes encoding L23 (Niben101Scf01444g02009) and FBOX (Niben101Scf04495g02005) were selected as constitutive internal controls for *N*. *benthamiana* genes [65]. Six biological replicates of the entire experiment were performed. Gene expression was normalized with respect to constitutively expressed internal controls, quantified and plotted using R software.

### Semi-quantitative RT-PCR

RNA extraction, cDNA synthesis and PCR were performed as described in [62]. Primer sequences are shown in S4 Table.

### Immuno-blot

Infiltrated *N*. *benthamiana* leaves were sampled at 48 hpa. 100 mg (fresh weight) of plant tissue were ground in 200 uL of Laemmli buffer, boiled and centrifuged 5min at 13 000 rpm. 75 µl of the supernatant were separated on a 12% polyacrylamide gel and transferred onto a nitrocellulose membrane by wet transfer. The membrane was blocked for 30 min with buffer (PBS 1X, Tween 1% and 5% skimmed milk), then incubated with 1/30 000 anti-GFP overnight. Next day, the membrane was washed 3 times 10 min with buffer (PBS 1X, Tween 0.5%) and incubated 4 hours with 1/25000 anti-GAR-PO antibodies (Goat Anti-rabbit Peroxidase). Proteins were detected with ECL using a G:Box (Syngene) imaging system. Finally, the membrane was stained with Ponceau red.

### BFA assays

A suspension of *A*. *tumefaciens* cells at OD600 of 0.2 carrying a construct for constitutive expression of either GFP, PR1sp:GFP or Pv33sp:GFP was infiltrated on the abaxial side of *N*. *benthamiana* leaves using a syringe without a needle. Twenty-four hours after agroinfiltration, a solution of 10 µM BFA in agroinfiltration medium (10 mM MgCl2, 10 mM MES pH 7.5) was infiltrated on the same spots. Samples were collected and used for confocal imaging after 4 hours.

### Imaging

White light images from *V*. *vinifera* and *N*. *benthamiana* leaves were taken at 6 dpa using a Nikon D5000.

Blue light images from *N*. *benthamiana* leaves were taken with a G:Box (Syngene) imaging system with blue LED at 465 nm; except for Fig 8A which was taken with white and blue LED at 465 nm.

Epifluorescence microscopy of *V*. *vinifera* leaves was performed with a Zeiss Axio Imager M2 microscope. Images were taken under Green fluorescent protein (GFP) excitations at 470 nm.

Confocal microscopy images were obtained with a LSM700 confocal laser microscope (Carl Zeiss, Jena, Germany), using a 20X water-immersion objective lens. GFP fluorescence was observed after excitation at 488 nm, with a 527 short-pass emission filter.

## Supporting information

S1 Fig WY-domain-containing proteins carrying dEER motifs are found in several oomycete species. Alignment of the N-terminal protein sequences of candidate WY-domain-containing proteins carrying EER motifs from *Phytophthora infestans* **(A)** *P*. *parasitica* **(B)** *Peronospora tabac*ina **(C)** *Plasmopara halstedii* **(D, E)** and *Hyaloperonospora arabidopsidis* **(F)**.
Red asterisks show proteins described as containing WY-domains in Derevnina et al. 2015 (C), Sharma et al. 2015 (D), Pecrix et al. 2019 (E). All P. infestans proteins and proteins with blue asterisks are annotated as RxLR or RXLR-like. Red boxes show signal peptides with red lines indicating cleavage site. Green boxes indicate EER motifs. Alignments were performed with MUSCLE, manually edited and displayed with BOXshade with a cutoff of 60% of sequences identical for shading. The procedure for the identification of the displayed proteins is described in S1 File.
(TIF)

S2 Fig Cell death induction following transient expression of Pv33ΔSP in grapevine leaves using a syringe. **(A)** Grapevine leaf infiltrated with 33ΔSP, 33FL, MYBA1, and GUS (negative control). VvMYBA1 is a transcription factor involved in anthocyane biosynthesis that allows verifying the efficiency of the transformation by the apparition of red colour. **(B)** Close-up images from (A). Pictures taken at 6 days post-infiltration.
(TIF)

S3 Fig Protein sequence logo showing Pv33 variability. Variability of Pv33 in 7 *P*. *viticola* isolates. Signal peptide is coloured in blue and EER motif in green. Polymorphisms are shown in yellow and residues from the reference sequence in red. Conserved amino acids are shown in grey.
(TIF)

S4 Fig Selected *P*. *viticola* WY-domain secreted proteins do not suppress the INF1-mediated cell death in *N*. *benthamiana*. Agrobacterium strains containing the different clones were infiltrated in *N*. *benthamiana* leaves. One day later, INF1 was transiently expressed in the infiltrated patches. Pictures are taken 5 days after INF1 agrobacterium-mediated infiltration. GUS as Avr3A are used as respectively as negative and positive control for INF1-mediated cell death suppression.
(TIF)

S5 Fig Habitus of *N*. *benthamiana* TRV-silenced plants used in this study. Pictures were taken 3 weeks after agroinfiltration with *A*. *tumefaciens* cells carrying a construct for silencing of *uidA*, *EDS1* or *SGT1*.
(TIF)

S6 Fig *P*. *viticola* WY-domain-containing proteins conserved among oomycetes. **(A)** Alignment of proteins from different oomycetes showing similarity to the *P*. *viticola* WY-domain-containing protein Pv221r1_s0028g11635. PHALS_07224: *Plasmopara halstedii*; PPTG_04390: *Phytophthora parasitica*; PITG_15032: *P*. *infestans*; PHYSODRAFT_2862: *P*. *sojae*; Hyalo: *Hyaloperonospora arabidopsidis*, JH597989.1_525858_528829_+; Ptabac: *Peronospora tabacina*, NBSG01000057.1_73227_75607_+. **(B)** Alignment of proteins from different oomycetes showing similarity to Pv33. PCACT: *Phytophthora cactorum*, RAW42459.1; PPAT: *P*. *parasitica*, ETI42875.1; PPALM: *P*. *palmivora*, POM73314.1; PLHAL: *Plasmopara halstedii*, CEG39835.1; PSOJ: *P*. *sojae*, XP_009539115.1; PMEG: *P*. *megakarya*, OWZ10859.1; PINF: *P*. *infestans*, XP_002900344.1.
Red boxes show signal peptides with red lines indicating cleavage site. Green boxes indicate EER motifs. Blue boxes show the position of the RxLR motifs in proteins from *Phytophtora* spp. Alignment performed with ClustalW and displayed with BOXshade, with a cutoff of 60% of sequences identical for shading. The final part of the alignments is not shown for the sake of clarity.
(TIF)

S7 Fig Pv33 protein sequence and alignment of the N-terminus of WY-domain proteins from the same clade as Pv33. **(A)** Pv33 protein sequence. Signal peptide, dEER and WY motifs are respectively highlighted in blue, red and green. **(B)** Alignment of the N-terminus of the WY-domain proteins from clade X in Fig 2. Signal peptide (SP) and dEER motifs are highlighted. Alignment performed with ClustalW and displayed with BOXshade, with a cutoff of 70% of sequences identical for shading.
(TIF)

S8 Fig Semi-quantitative RT-PCR of Pv33 expression in *P*. *viticola* developmental stages. RT-PCR with 35 cycles of amplification in sporangia (Sp), germinated spores (Sg) and infected tissues at 0, 24, 48 and 72 hours after inoculation (hpi).
(TIF)

S1 Table Summary of results of the search for proteins similar to *P*. *viticola* WY-domain-containing proteins in other oomycetes. (PDF)

S2 Table Results of amplification of the Pv33 sequence in *P*. *viticola* isolates. Results of PCR amplification of Pv33 on genomic DNA from P. viticola isolates from different geographical origins and host sources. PCRs were performed using primers designed to amplify the whole gene sequence, from ATG to STOP. PCR with primers for the P. viticola tubulin gene confirmed the presence of pathogen DNA in all samples.
(PDF)

S3 Table RNA-Seq statistics and RSA accession numbers. (PDF)

S4 Table Constructs and primers used in this study. (PDF)

S1 File Supplementary materials and methods. (PDF)

S1 Dataset Protein sequences of the 68 WY-domain proteins containing dEER motifs. (TXT)

S2 Dataset Database of oomycete RXLR effectors. (TXT)

S3 Dataset RNA-Seq data. (XLSX)
