# Supplementary figures and images for: A secreted WY-domain-containing protein present in European isolates of the oomycete Plasmopara viticola induces cell death in grapevine and tobacco species
Source: PLoS One. 2019 Jul 29;14(7):e0220184. doi: 10.1371/journal.pone.0220184 (PMC6663016; doi:10.1371/journal.pone.0220184)

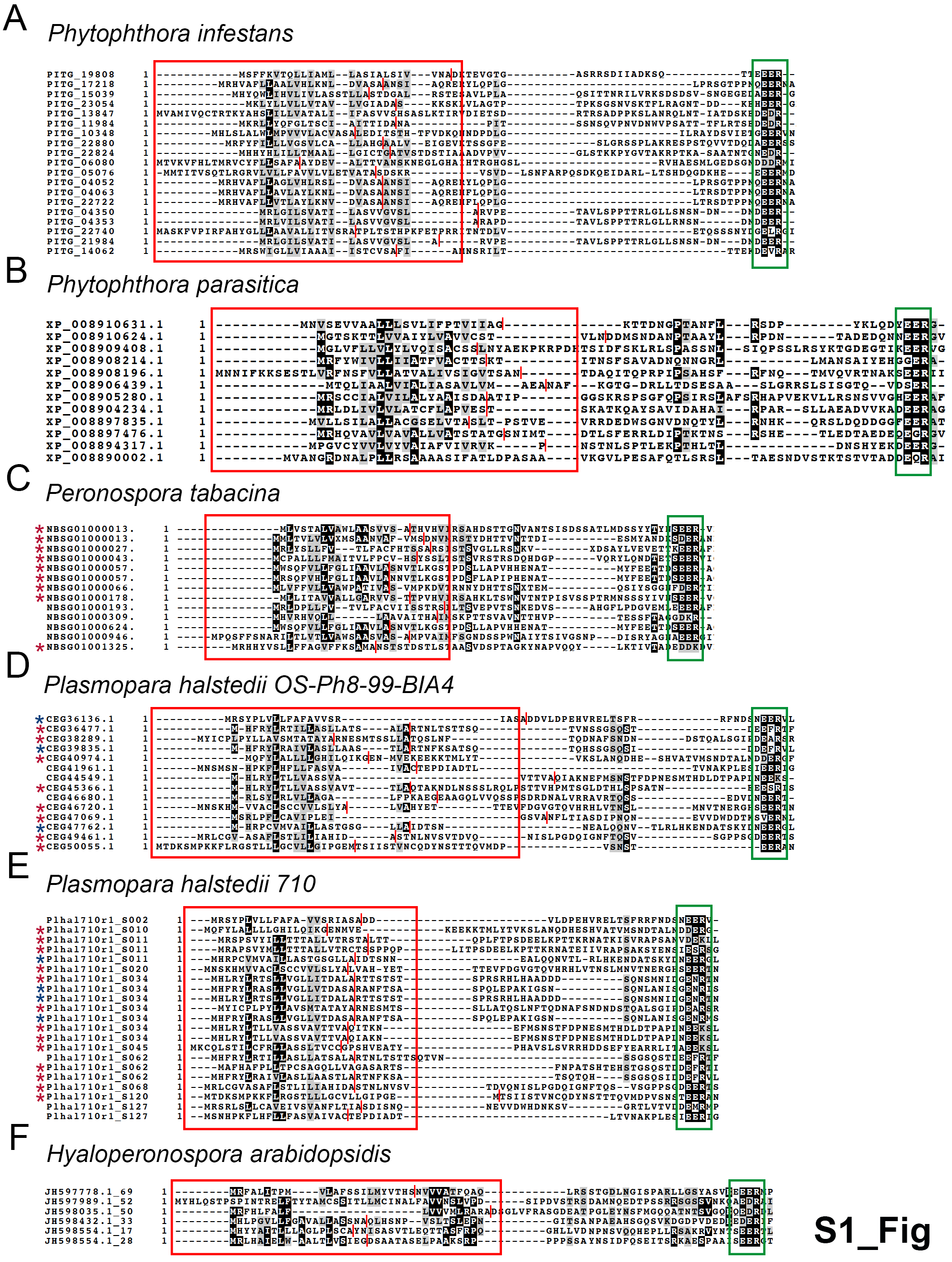

Supplement: S1 Fig — Alignment of the N-terminal protein sequences of candidate WY-domain-containing proteins carrying EER motifs from Phytophthora infestans (A) P. parasitica (B) Peronospora tabacina (C) Plasmopara halstedii (D, E) and Hyaloperonospora arabidopsidis (F). Red asterisks show proteins described as containing WY-domains in Derevnina et al. 2015 (C), Sharma et al. 2015 (D), Pecrix et al. 2019 (E). All P. infestans proteins and proteins with blue asterisks are annotated as RxLR or RXLR-like. Red boxes show signal peptides with red lines indicating cleavage site. Green boxes indicate EER motifs. Alignments were performed with MUSCLE, manually edited and displayed with BOXshade with a cutoff of 60% of sequences identical for shading. The procedure for the identification of the displayed proteins is described in S1 File. (TIF) [file pone.0220184.s001.tif]

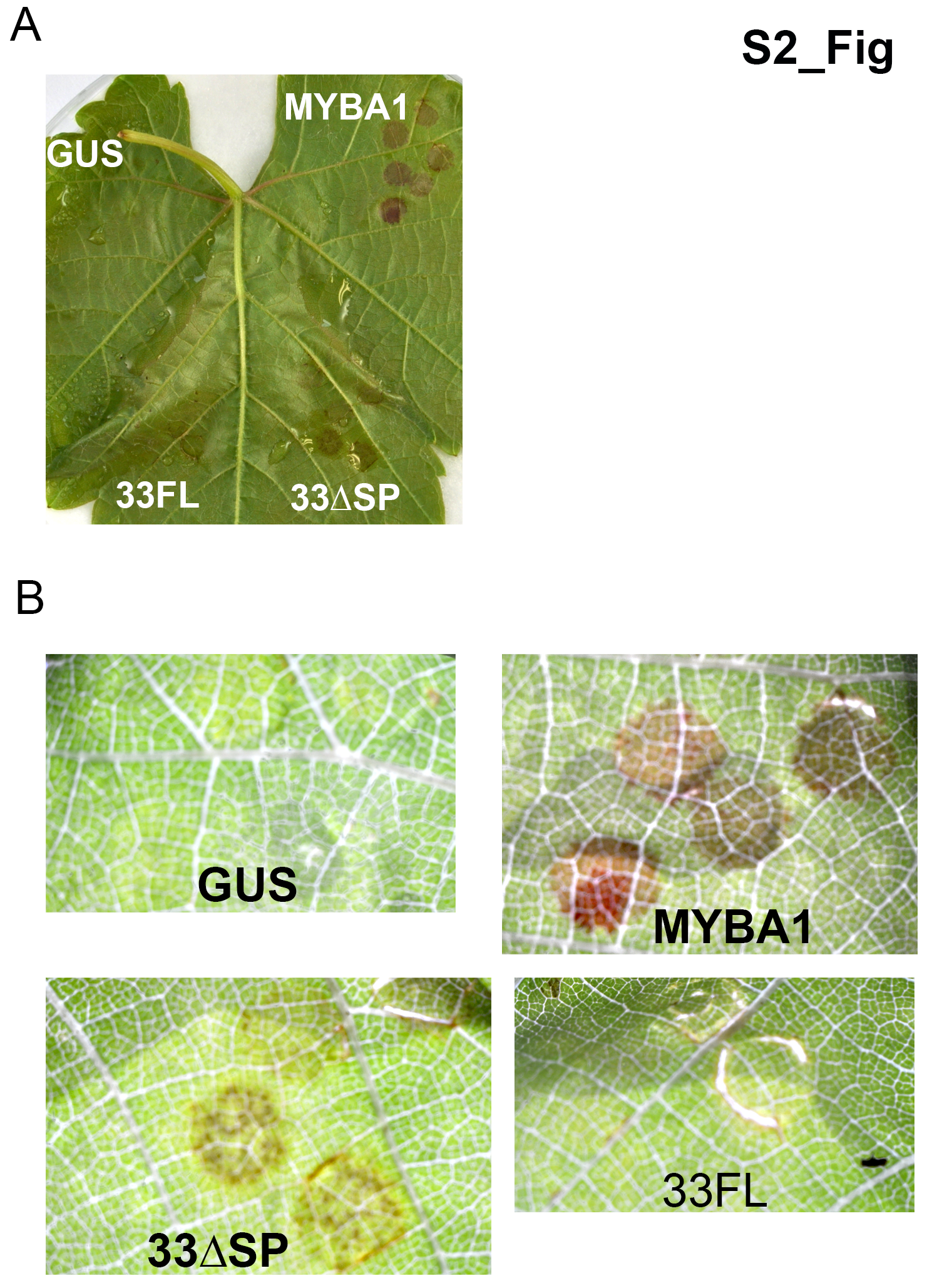

Supplement: S2 Fig — (A) Grapevine leaf infiltrated with 33ΔSP, 33FL, MYBA1, and GUS (negative control). VvMYBA1 is a transcription factor involved in anthocyane biosynthesis that allows verifying the efficiency of the transformation by the apparition of red colour. (B) Close-up images from (A). Pictures taken at 6 days post-infiltration. (TIF) [file pone.0220184.s002.tif]

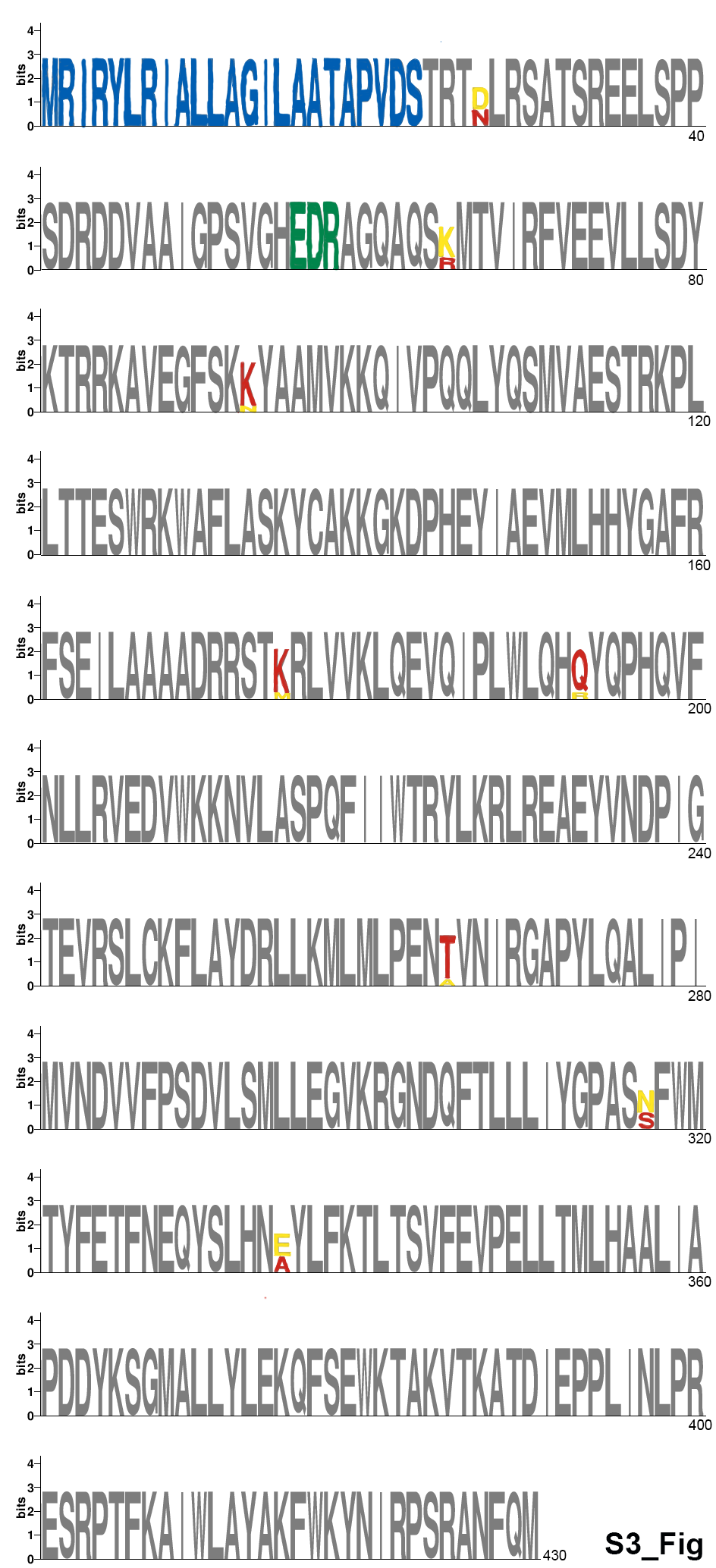

Supplement: S3 Fig — Variability of Pv33 in 7 P. viticola isolates. Signal peptide is coloured in blue and EER motif in green. Polymorphisms are shown in yellow and residues from the reference sequence in red. Conserved amino acids are shown in grey. (TIF) [file pone.0220184.s003.tif]

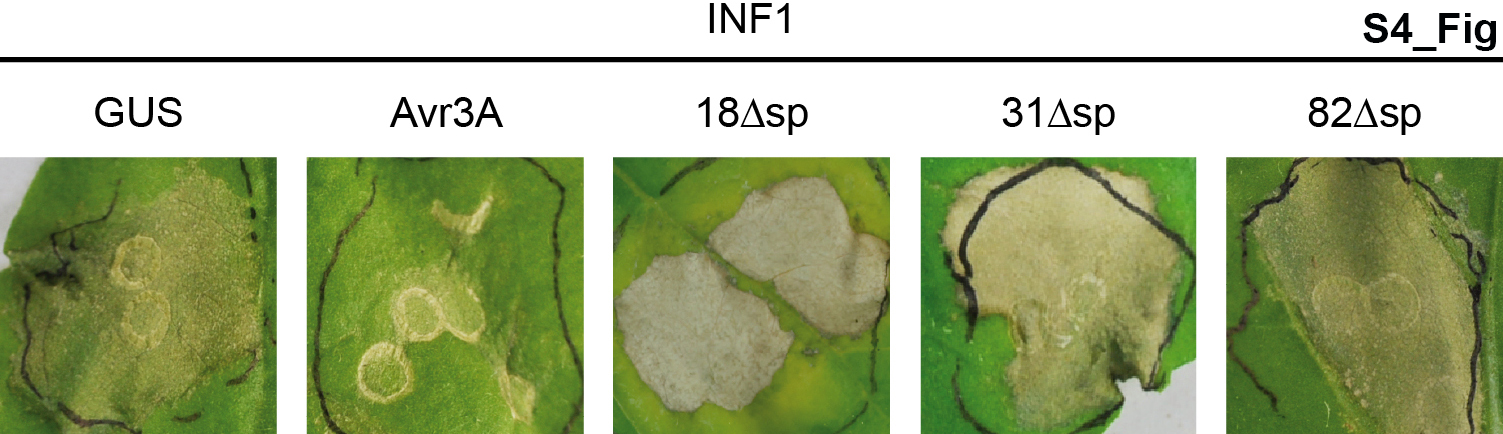

Supplement: S4 Fig — Agrobacterium strains containing the different clones were infiltrated in N. benthamiana leaves. One day later, INF1 was transiently expressed in the infiltrated patches. Pictures are taken 5 days after INF1 agrobacterium-mediated infiltration. GUS as Avr3A are used as respectively as negative and positive control for INF1-mediated cell death suppression. (TIF) [file pone.0220184.s004.tif]

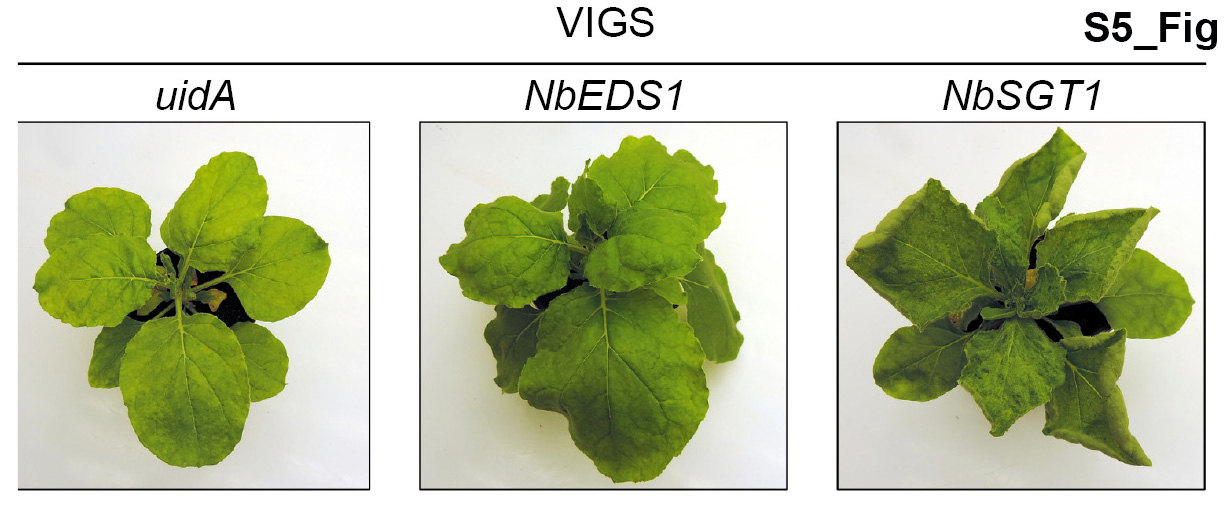

Supplement: S5 Fig — Pictures were taken 3 weeks after agroinfiltration with A. tumefaciens cells carrying a construct for silencing of uidA, EDS1 or SGT1. (TIF) [file pone.0220184.s005.tif]

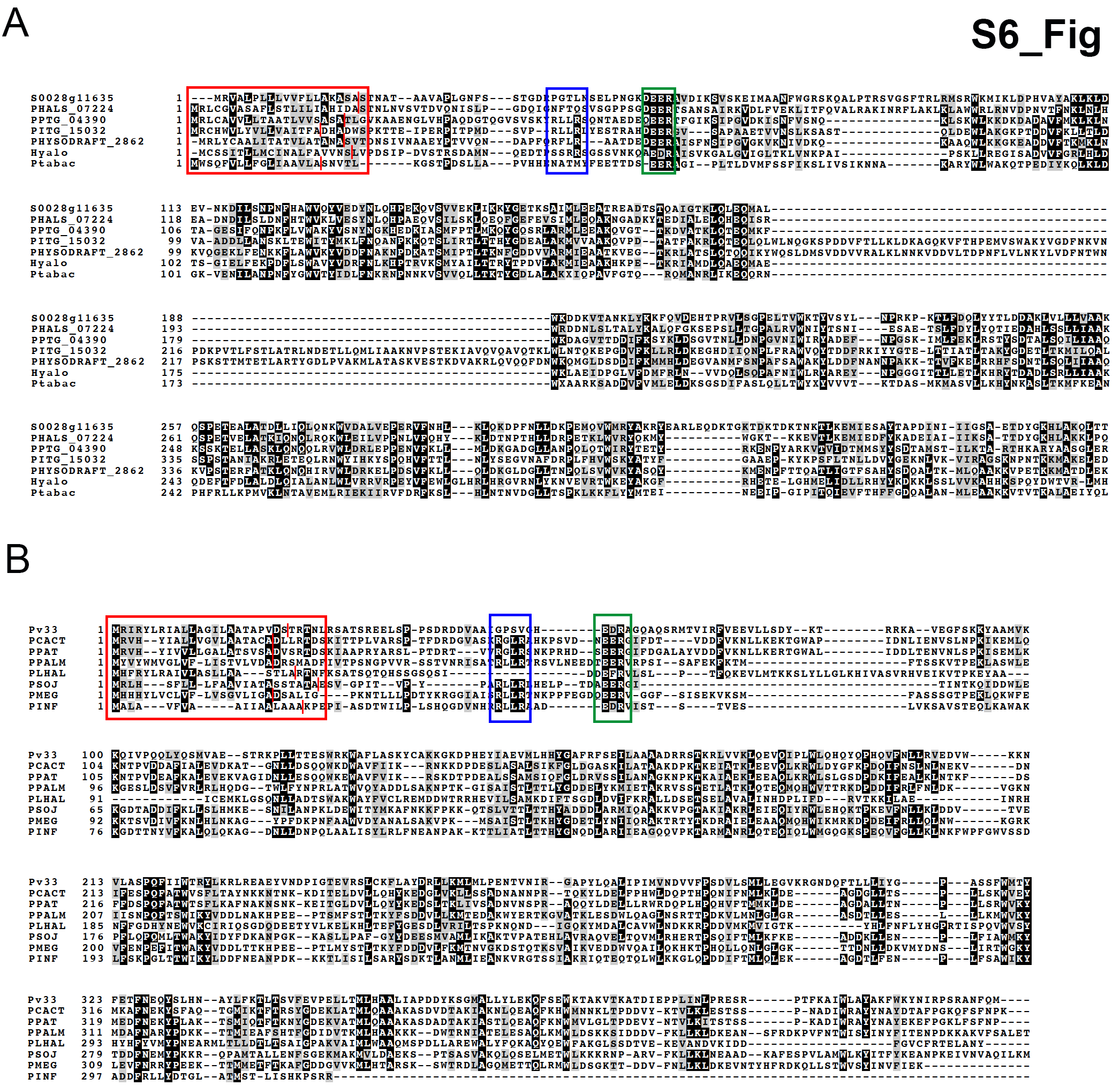

Supplement: S6 Fig — (A) Alignment of proteins from different oomycetes showing similarity to the P. viticola WY-domain-containing protein Pv221r1_s0028g11635. PHALS_07224: Plasmopara halstedii; PPTG_04390: Phytophthora parasitica; PITG_15032: P. infestans; PHYSODRAFT_2862: P. sojae; Hyalo: Hyaloperonospora arabidopsidis, JH597989.1_525858_528829_+; Ptabac: Peronospora tabacina, NBSG01000057.1_73227_75607_+. (B) Alignment of proteins from different oomycetes showing similarity to Pv33. PCACT: Phytophthora cactorum, RAW42459.1; PPAT: P. parasitica, ETI42875.1; PPALM: P. palmivora, POM73314.1; PLHAL: Plasmopara halstedii, CEG39835.1; PSOJ: P. sojae, XP_009539115.1; PMEG: P. megakarya, OWZ10859.1; PINF: P. infestans, XP_002900344.1. Red boxes show signal peptides with red lines indicating cleavage site. Green boxes indicate EER motifs. Blue boxes show the position of the RxLR motifs in proteins from Phytophtora spp. Alignment performed with ClustalW and displayed with BOXshade, with a cutoff of 60% of sequences identical for shading. The final part of the alignments is not shown for the sake of clarity. (TIF) [file pone.0220184.s006.tif]

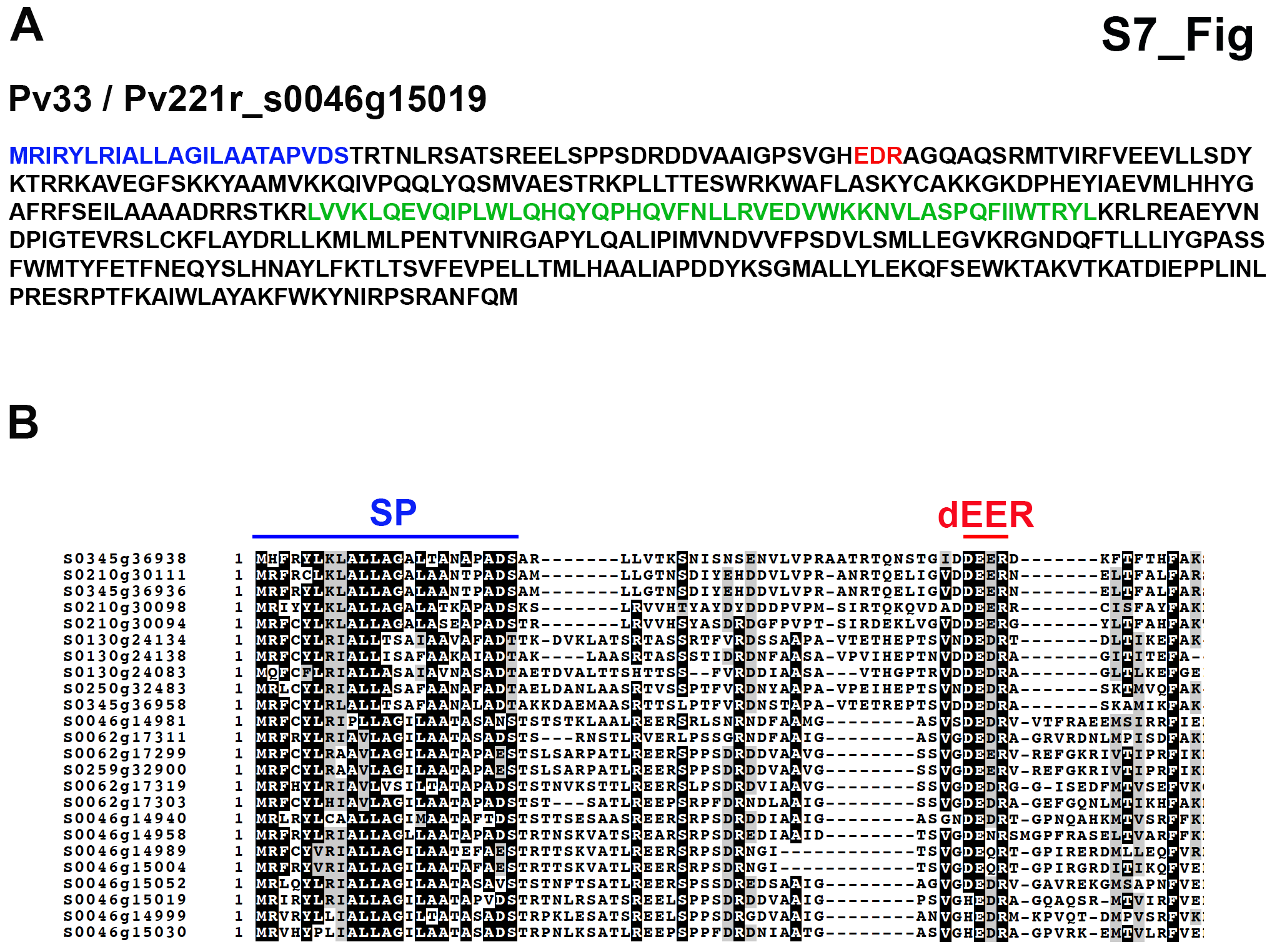

Supplement: S7 Fig — (A) Pv33 protein sequence. Signal peptide, dEER and WY motifs are respectively highlighted in blue, red and green. (B) Alignment of the N-terminus of the WY-domain proteins from clade X in Fig 2. Signal peptide (SP) and dEER motifs are highlighted. Alignment performed with ClustalW and displayed with BOXshade, with a cutoff of 70% of sequences identical for shading. (TIF) [file pone.0220184.s007.tif]

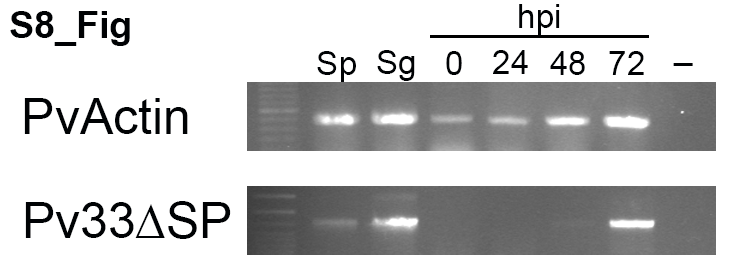

Supplement: S8 Fig — RT-PCR with 35 cycles of amplification in sporangia (Sp), germinated spores (Sg) and infected tissues at 0, 24, 48 and 72 hours after inoculation (hpi). (TIF) [file pone.0220184.s008.tif]
