# Supplementary material for: A secreted WY-domain-containing protein present in European isolates of the oomycete Plasmopara viticola induces cell death in grapevine and tobacco species
Source: PLoS One. 2019 Jul 29;14(7):e0220184. doi: 10.1371/journal.pone.0220184 (PMC6663016; doi:10.1371/journal.pone.0220184)
Supplement: S1 Table — (PDF) [file pone.0220184.s009.pdf]

Supplementary Table 1. Summary of results of the search for proteins similar to *P. viticola* WY-domain-containing proteins in other oomycetes.

Species	Total hits[a]	RXLR[b]	No RXLR[c]	No RXLR – EER[d]
Phytophthora infestans	87	66	21	19
Phytophthora parasitica	63	51	12	12
Peronospora tabacina	25	10	15	13
Plasmopara halstedii 710	35	4	31	21
Plasmopara halstedii OS-Ph8-99-BIA4	20	2	18	14
Hyaloperonospora arabidopsidis	6	0	6	6

a: Blast hits after filtering for presence of signal peptide and absence of transmembrane domain. **b**: Number of proteins from *a* carrying RXLR or RXLR-like motifs. **c**: Number of proteins from *a* lacking RXLR or RXLR-like motifs. **d**: Number of proteins from *c* showing dEER or dEER-like motifs inside the first 80 amino acids.

Details of Blast searches and filtering are described in the Supplementary Methods section.
