# Supplementary material for: A secreted WY-domain-containing protein present in European isolates of the oomycete Plasmopara viticola induces cell death in grapevine and tobacco species
Source: PLoS One. 2019 Jul 29;14(7):e0220184. doi: 10.1371/journal.pone.0220184 (PMC6663016; doi:10.1371/journal.pone.0220184)
Supplement: S2 Table — Results of PCR amplification of Pv33 on genomic DNA from P. viticola isolates from different geographical origins and host sources. PCRs were performed using primers designed to amplify the whole gene sequence, from ATG to STOP. PCR with primers for the P. viticola tubulin gene confirmed the presence of pathogen DNA in all samples. (PDF) [file pone.0220184.s010.pdf]

Supplementary Table 2. Results of PCR amplification of Pv33 on genomic DNA from P. viticola isolates from different geographical origins and host sources. PCRs were performed using primers designed to amplify the whole gene sequence, from ATG to STOP. PCR with primers for the P. viticola tubulin gene confirmed the presence of pathogen DNA in all samples.

Isolate	Host	Collection site	Amplification
PvSC	*V. vinifera*	Colmar, France	✓
Pv393	*V. vinifera*	Cugnasco, Switzerland	✓
Pv340	*V. vinifera*	Tolcsva, Hungary	✓
Pv257	*V. vinifera*	Listrac, France	✓
Pv125	Regent	Pécs, Hungary	✓
Pv412	Regent	Cugnasco, Switzerland	✓
Pv13	Regent	Latresne, France	✓
MSU1106	*V. vinifera*	Clarksville, Michigan, USA	✗
NY220	*V. vinifera*	Long Island, New York, USA	✗
MSU1052	*V. labrusca*	Fenville, Michigan, USA	✗
MSU319	*V. labrusca*	Stones, Ohio, USA	✗
MSU330	*V. aestivalis*	Markko, Ohio, USA	✗
WV16	*V. aestivalis*	Convington, West Virginia, USA	✗
MSU162	*V. vinifera*	Jackson, Michigan, USA	✗
FLO23	*V. aestivalis*	Thallahassee, Florida, USA	✗
NY328	*V. riparia*	Ithaca, New York, USA	✗
MSU1172	*V. riparia*	Fennville, Michigan, USA	✗
