# Supplementary material for: A secreted WY-domain-containing protein present in European isolates of the oomycete Plasmopara viticola induces cell death in grapevine and tobacco species
Source: PLoS One. 2019 Jul 29;14(7):e0220184. doi: 10.1371/journal.pone.0220184 (PMC6663016; doi:10.1371/journal.pone.0220184)
Supplement: S3 Table — (PDF) [file pone.0220184.s011.pdf]

Supplementary Table 3. RNA-Seq statistics and RSA accession numbers.

Provider	Sample	Biological replicate	Host	Tissue	# of pairs	size (nt)	# of unambiguous mapped pairs on Pv221 genome	SRA accession
INRA / GET-Plage	Pv221 24hpi	1	Vitis vinifera cv. Muscat Ottonel	leaves	46 047 551	100	16 670	SRR7500372
INRA / GET-Plage	Pv221 24hpi	2	Vitis vinifera cv. Muscat Ottonel	leaves	31 978 558	100	29 439	SRR7500373
INRA / GET-Plage	Pv221 24hpi	3	Vitis vinifera cv. Muscat Ottonel	leaves	26 751 573	100	19 740	SRR7500375
INRA / GET-Plage	Pv221 48hpi	1	Vitis vinifera cv. Muscat Ottonel	leaves	48 442 856	100	1 294 356	SRR7500376
INRA / GET-Plage	Pv221 48hpi	2	Vitis vinifera cv. Muscat Ottonel	leaves	53 270 151	100	307 908	SRR7500369
INRA / GET-Plage	Pv221 48hpi	3	Vitis vinifera cv. Muscat Ottonel	leaves	30 664 708	100	227 777	SRR7500370
INRA / GET-Plage	Pv221 72hpi	1	Vitis vinifera cv. Muscat Ottonel	leaves	31 057 365	150	369 013	SRR7500374
INRA / GET-Plage	Pv221 72hpi	2	Vitis vinifera cv. Muscat Ottonel	leaves	37 993 907	150	1 314 505	SRR7500371
INRA / GET-Plage	Pv221 72hpi	3	Vitis vinifera cv. Muscat Ottonel	leaves	36 069 269	150	304 513	SRR7500377
