# Supplementary material for: A secreted WY-domain-containing protein present in European isolates of the oomycete Plasmopara viticola induces cell death in grapevine and tobacco species
Source: PLoS One. 2019 Jul 29;14(7):e0220184. doi: 10.1371/journal.pone.0220184 (PMC6663016; doi:10.1371/journal.pone.0220184)
Supplement: S4 Table — (PDF) [file pone.0220184.s012.pdf]

Supplementary Table 4. Constructs and primers used in this study.

Gene identities

Pv18	Plvit221r1_S0028g11635
Pv31	Plvit221r1_S0259g32900
Pv33	Plvit221r1_S0046g15019
Pv82	Plvit221r1_S0028g11682

Constructs

18Δsp	35S:$Pv18_{\Delta 1\text{-}19}$
31Δsp	35S:$Pv31_{\Delta 1\text{-}23}$
33Δsp	35S:$Pv33_{\Delta 1\text{-}23}$
82Δsp	35S:$Pv82_{\Delta 1\text{-}22}$
33FL	35S:Pv33
33Δsp-GFP	35S:$Pv33_{\Delta 1\text{-}23}$-GFP
33FL-GFP	35S:Pv33-GFP
33Δsp-GFP-NES	35S:$Pv33_{\Delta 1\text{-}23}$-GFP-NES
33Δsp-GFP-nes	35S $Pv33_{\Delta 1\text{-}23}$-GFP-nes

Primers for cloning in binary plasmids (restriction sites underlined)

PvRXL18sp	5’ GCCAAATCTAGAATGTCTACGAATGCGACTGC
PvRXL18R	5’ TAGAAGCCCGGGCTATGCTGGTGCGGTGGT
PvRXL31sp	5’ GCACCCTCTAGAATGACAAGTCTGAGTGCC
PvRXL31R	5’ ACAACACCCGGGTCACATTACAAAATCCTC
PvRXL33F	5’ TCATTCTCTAGAATGCGCATTCGCTACTTG
PvRXL33sp	5’ GCACCCTCTAGAATGACAAGGACGAATCTTCG
PvRXL33R	5’ CTCTTGCCCGGGTCACATTTGAAAATTCGC
PvRXL33gfF	5’ TCATTCGTCGACATGCGCATTCGCTACTTG
PvRXL33gfsp	5’ GCACCCGTCGACATGACAAGGACGAATCTTCG
PvRXL33gfR	5’ CTCTTGTCTAGACATTTGAAAATTCGCTC
PvRXL82sp	5’ GCAAAATCTAGAATGTCTGCGAATGCGACAGCA
PvRXL82R	5’ ACTAGTCCCGGGCTACAACTCCTCTGCCAG
GFP-F	5’ GACTAGTCTAGAATGAGTAAAGG

GFPNES
5’ GCAAACcccgggCTACTTGTTAATATCAAGTCCAGCCAACTTAAGAGCAAGCTCGTTtttgtatagttcatccatg
GFPnes
5’ GCAAACcccgggCTACTTGTTAGCATCTGCTCCAGCTGCCTTAAGAGCAAGCTCGTTtttgtatagttcatccatg

Primers for V. vinifera HSR and Actin for RT-PCR

VvACT-F	5’ GTGCCAATTTATGAAGGTTATGC
VvACT-R	5’ CCCTCTCAGTTAGAATCTTCATCAG
VvHSR-F	5’ GGACTACCGACATGCACCTG
VvHSR-R	5’ CCTGGACAATTCTGCCATCT
