# Supplementary material for: A secreted WY-domain-containing protein present in European isolates of the oomycete Plasmopara viticola induces cell death in grapevine and tobacco species
Source: PLoS One. 2019 Jul 29;14(7):e0220184. doi: 10.1371/journal.pone.0220184 (PMC6663016; doi:10.1371/journal.pone.0220184)
Supplement: S1 File — (PDF) [file pone.0220184.s013.pdf]

Supplementary Material and Methods

Search for proteins similar to *P. viticola* WY-domain-containing proteins in other oomycetes.
The 68 WY-domain-containing proteins from *P. viticola* were used as a query in a BLAST search at low stringency (Evalue<10e-5) against the proteomes of *Phytophthora infestans* T30-4 (PRJNA17665; Haas et al, 2009), *Phytophthora parasitica* INRA-310 (PRJNA259235), *Peronospora tabacina* 968-S26 (PRJNA285243; Derevnina et al, 2015), *Plasmopara halstedii* OS-Ph8-99-BIA4 (PRJEB6932; Sharma et al, 2015), *Plasmopara halstedii* 710 (PRJNA412027; Pecrix et al, 2019) and *Hyaloperonospora arabidopsidis* Emoy2 (PRJNA30969; Baxter et al, 2010). The BLAST output was analyzed with SignalP4 and TMHMM 2.0, and proteins lacking a signal peptide or possessing a transmembrane domain outside the first 70 amino acids were discarded. Remaining proteins were aligned with MUSCLE and proteins carrying RXLR or RXLR-like motifs were identified by visual inspection of the alignments. The presence of dEER or dEER-like motifs was determined based on the visual inspection of alignments of proteins lacking RXLR motifs. Alignments in Supplementary Figure 1 show proteins lacking RXLR or RXLR-like motif that possess dEER or dEER-like motifs.

Transient expression of 33ΔSP in grapevine leaves using a syringe.
Agrobacterium suspensions were prepared as described in the manuscript, excepting the addition that Silwet-L77. Infiltrations were performed with a needleless 1 ml syringe as described in Zottinni et al. (2008). The infiltration was limited to the area of the leaf in contact with the syringe.

References
Haas B, Kamoun S, Zody M, Jiang R, Handsaker R, et al. (2009) Genome sequence and analysis of the Irish potato famine pathogen *Phytophthora infestans*. Nature 461: 393–398.

Zottini, M. *et al.* (2008) Agroinfiltration of grapevine leaves for fast transient assays of gene expression and for long-term production of stable transformed cells. *Plant Cell Reports* **27**, 845-853.
